# Decoding Trap States in Working 2D Perovskite Multi‐Functional Devices

**DOI:** 10.1002/advs.202518675

**Published:** 2026-01-12

**Authors:** Ioannis Leontis, Karl Jonas Riisnaes, Hoi Tung Lam, Rosanna Mastria, Luisa De Marco, Annalisa Coriolano, Steven Hepplestone, Monica Felicia Craciun, Saverio Russo

**Affiliations:** ^1^ Centre for Graphene Science, College of Engineering, Mathematics and Physical Sciences University of Exeter Exeter EX4 4QL UK; ^2^ CNR NANOTEC Institute of Nanotechnology via Monteroni 73100 Lecce Italy

**Keywords:** 2D perovskites, defects, multi‐functional devices, trap states spectroscopy

## Abstract

Hybrid organic–inorganic perovskites, such as 4‐fluorophenethylammonium lead iodide (F‐PEAI), is an emerging class of layered 2D materials of unique interest for a range of opto‐electronic applications, owing to their exceptional photophysical properties and enhanced environmental stability. Yet, the device performance is strongly governed by trap states, which remain challenging to characterise under realistic operating conditions. This is even more prominent in multi‐functional devices where, according to the user‐defined dominant charge carrier type and traps dynamic the system switches from a field effect transistor (FET), to a highly sensitive photodetectors, energy harvesting or a even synaptic device. Characterising trap states for each of these regimes is uniquely challenging as it requires methods capable of distinguishing different types of carriers and trapping centers within the stringent operating requirements, and ideally without compromising the functionalities of the device. Here, threshold voltage transient spectroscopy (TVTS) is introduced as a universal, non‐invasive method to probe trap states in fully processed 2D F‐PEAI single‐crystal field‐effect transistors that also function as high‐gain photodetectors. TVTS enables real‐time extraction of sub‐gap trap densities and energy distributions, with tunable sensitivity to emission or retrapping processes at any set temperature, providing insight into their real‐time influence on charge transport mechanisms. When applied to 2D F‐PEAI multi‐functional devices, TVTS reveals a transition in trap states and dynamics from deep majority‐carrier trapping (10^13^ cm^−2^) at cryogenic temperature to shallow trapping above 100K. Strong retrapping is observed to enhance minority‐carrier diffusion lengths ( 5 µm), yielding responsivities up to 120 A/W providing a pathway for enhancing the opto‐electronic device performance. These results establish TVTS as a powerful platform for in situ defect spectroscopy of multifunctional 2D perovskite devices.

## Graphical abstract

Illustration of the photoluminescence spectrum and energy‐level landscape governing trap‐mediated carrier dynamics in 2D F‐PEAI photodetectors. A novel approach capturing dynamic trap occupation and retrapping in devices under operating conditions, directly links defect energetics to carrier transport and enhanced photoresponse in multifunctional perovskite devices.

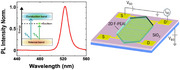

## 1 Introduction

Layered 2D hybrid organic/inorganic perovskites represent an emerging class of materials with exceptional photophysical properties, positioning them as prime candidates for applications in sensing, energy conversion, computing, and information technologies. These hybrid materials feature quantum confinement of charge carriers within self‐assembled quantum wells, defined by alternating inorganic layers and organic spacer molecules. This unique structural arrangement enables precise tailoring of their electrical and optical properties through compositional engineering,^[^ ^1^ ^]^ unlocking a broad range of optoelectronic applications, including solar cells, light‐emitting diodes, neuromorphic memories, and photodetectors. These unique properties have recently been shown to support the development of multi‐functional devices such as perovskite LED displays that can also function as touch screens, ambient light sensors, image sensors and energy harvesting.^[^ ^2^ ^]^ Similarly, 2D and 3D perovskite field effect transistors (FETs) with synaptic plasticity have been used in multi‐functional neuromorphic applications enabling object recognition and motion perception^[^ ^3^, ^4^ ^]^ or photoresponse to full‐spectrum optical pulse signals, which mimic biological neural networks.^[^ ^5^ ^]^ Another example is a multi‐functional self‐powered perovskite light sensors with the capability of advanced autonomous processing of health signals using reservoir computing.^[^ ^6^ ^]^ In these devices, each functionality is user‐defined and fully determined by the operating conditions ultimately setting the dominant charge carrier type, traps and overall charge dynamics. Interestingly, several of these functionalities leverage on contrasting charge carrier dynamics since many opto‐electronic devices (e.g. photodetectors and p‐n photodiodes) exploit the minority carriers dynamics whilst a vast number of electronic devices (e.g. FETs, memories, p‐i‐n solar cells and Schottky photo‐diodes) rely on the majority carriers. Understanding the interplay of these opposing carrier dynamics within a single multifunctional device presents a unique challenge, as it demands advanced methods capable of disentangling co‐existing charge transport processes and trapping centers, each operating within a narrow and overlapping range of functional parameters.

To date, light‐assisted methods have shown promise for studying layered perovskites,^[^ ^7^, ^8^, ^9^, ^10^, ^11^ ^]^ however, these generally struggle to probe non‐radiative defects far beyond the interfacial region due to high optical density materials.^[^ ^12^ ^]^ At the same time, transient photocurrent (PICTS) has been shown to be provide information primarily limited to the minority carrier dynamics, and mainly when thermal‐assisted emission of the trapped carriers is the dominant mechanism during the detrapping process^[^ ^13^ ^]^ making it unsuitable to unveil the complex dynamics underpinning emerging multi‐functional devices. Furthermore, light‐assisted methods generally struggle to probe non‐radiative defects beyond the light absorption length region due to high optical density materials.^[^ ^7^, ^8^, ^9^, ^10^, ^11^, ^12^ ^]^ Finally, optical techniques are primarily sensitive to radiative transitions near the Brillouin zone center, thereby missing non‐radiative processes and the dynamics of trap states that interact with high‐momentum carriers (e.g. under bias), as encountered in transistors and other electronic devices under operating conditions.^[^ ^14^ ^]^ At the same time, whilst conventional pure electrical or opto‐electrical steady‐state and transient spectroscopies have been successfully employed to study defect states in bulk 3D perovskites,^[^ ^14^, ^15^, ^16^, ^17^, ^18^ ^]^ these techniques are generally not suitable to 2D systems where the confining layer of the charges is significantly smaller than the Debye screening. Indeed, traditional deep‐level trap spectroscopy (DLTS) and thermal admittance spectroscopy (AS) probe the concentration and dymanic of traps in the depleted area of the 3D semiconductor, which is not present in 2D systems. In addition, the reliance on AC admittance techniques effectively renders these techniques not suitable for probing slow trap state dynamics, such as those relevant for memory applications. Finally, most of these techniques require temperature‐dependent measurements to identify the energy depth of the trapping states, and distinguish between shallow and deep levels. This is incompatible with the wide range of organic/inorganic 2D systems whose stable crystal phases exist only within a narrow temperature windows. It is therefore essential to establish a method capable of characterizing the complex defect landscape and charge carrier dynamics in multifunctional 2D perovskite devices under tightly constrained operating conditions.

To this end, the recently developed threshold voltage transient spectroscopy (TVTS) for 2D transition metal dichalcogenides^[^ ^19^, ^20^ ^]^ offers significant advantages. As an electrically‐driven transient technique, it uses back gate field effect to inject carriers into the channel and to fill the inter‐gap trapping energy states enabling easy control on the energy range of the probed trapping centers provide information on both majority and minority carriers overcoming the limitations of photocurrent transient techniques such as PICTS. Additionally, TVTS permits very efficient tuning of the level of the injected carriers allowing probing of both pure emission dynamics and strong retrapping effects on the same device, depending on the device operational conditions without the need for using extreme high irradiance or bias to monitor retrapping effects as in transient photocurrent spectroscopy.^[^ ^21^ ^]^ Contrary to traditional deep‐level trap spectroscopy (DLTS) and admittance spectroscopy (AS), TVTS is uniquely suited to 2D systems in which the confinement of charges in a layer thinner than the Debye screening length ensures that the threshold voltage is strongly modulated by space charge regions at the semiconductor‐dielectric and semiconductor‐ambient interfaces, and it supports studies of trap states over a broad frequency range (down to <1 mHz, i.e. a thousand times lower than the bandwidth of DLTS and AS). Another key advantage of TVTS is that it can also distinguish deep from shallow traps from direct analysis of just a set of measurements at a specific temperature without the need for detailed Arrhenius plot studies, allowing real time characterization of the trapping effect and not just diagnoses of the trap dynamics. Additionally, TVTS probes the trap states simply using the electrical contacts utilised for the regular operation of devices, without the need to disassemble any parts and without causing any physical changes to device structures. Finally, since TVTS does not rely on the use of light for the filling of the traps, the method has no restrictions on the optical density of encapsulating layers, either. Building on these advancements, establishing a robust methodology for the in situ characterisation of defect states in multifunctional 2D perovskite devices under operating conditions would mark a significant step forward. Such an approach would enable targeted defect passivation, accelerate material screening, and facilitate real‐time quality control throughout the operational lifetime of multifunctional devices.

In this work, we pioneer the use of TVTS in a fully operational single‐crystal 2D 4‐fluorophenethylammonium lead iodide (F‐PEAI) perovskite field‐effect transistors, which also function as high‐gain photodetectors, providing critical insight into the impact of charge traps on the performance of this multi‐functional device. By studying the devices dynamics across a broad temperature range, from room temperature down to cryogenics (*T* = 4.2 K), and under various illumination conditions, we uncover the interplay between trap states and charge transport mechanisms. At temperatures *T* < 60 K, deep traps govern the charge carrier dynamics, with electrical transport in the 2D perovskite FETs driven by 2D variable range hopping (2D VRH). As the temperature increases, thermally activated band conduction (BC) emerges from *T* ⩾ 60 K, with shallow traps playing a key role in modulating conductivity and photoconductivity for *T* ⩾152 K. At room temperature, space‐charge‐limited photocurrent (SCLP) is observed, highlighting a high generation rate and long diffusion length of photoexcited carriers, along with unbalanced carrier transport within the 2D perovskite layers. With the aid of high‐resolution scanning photocurrent microscopy (SPCM) in planar photodetectors, we directly visualize the spatial diffusion of photoexcited charge carriers extending 5 µm beyond the direct source‐drain region under steady‐state conditions. In these devices, the interplay between defect states and Schottky barriers at the Au/2D perovskite contact interfaces lead to irradiance‐dependent figures of merit, underpinning the monotonic increase of photoresponsivity and external quantum efficiency upon decreasing irradiance. The non‐invasive characterization of charge traps in fully processed multi‐functional 2D perovskite devices unlocks the insight on their operational mechanisms and performance.

## 2 2D 4‐Fluorophenethylammonium Lead Iodide

**Figure 1:**
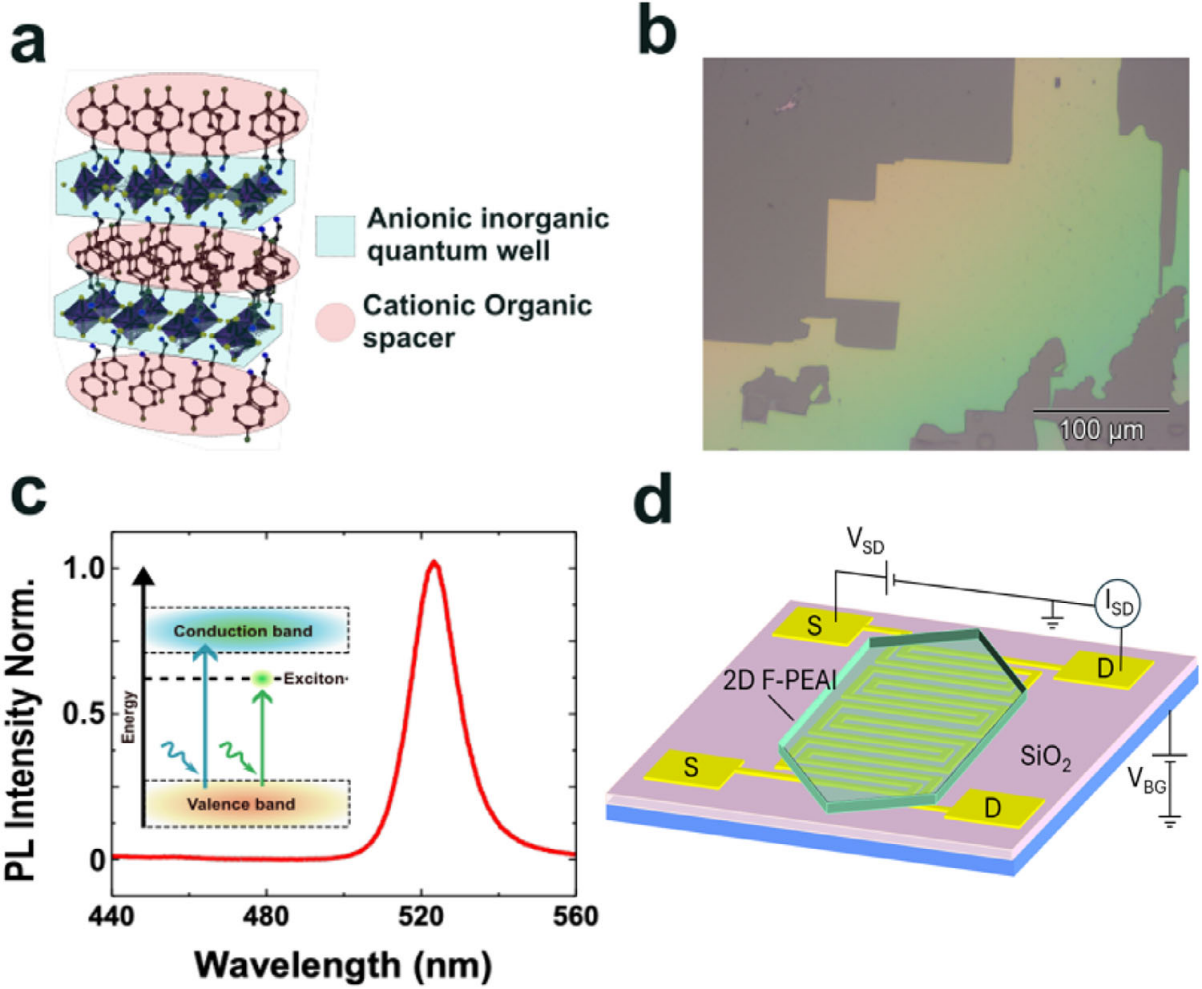
2D F‐PEAI crystal structure, optical characteristics and transistor/photodetector device and circuit diagram. a) Schematic of the 2D F‐PEAI crystal structure highlighting the [PbI_4_]^2 −^ octahedra and the organic spacing layer. b) White light optical micrograph of a 2D F‐PEAI crystals. c) Plot of the measured photoluminescence spectra of 2D F‐PEAI. The inset shows a schematic of the band‐to‐band and excitonic transitions. d) Schematic of the laminated 2D F‐PEAI crystal over the interdigitated Au electrodes fabricated on doped Si/SiO_2_ substrate and schematic of the circuit used for the characterisation of the FET.

Among the wide range of emerging 2D perovskites, F‐PEAI has attracted considerable attention due to its exceptional combination of environmental stability, processability, and optoelectronic performance of devices fabricated in ambient conditions.^[^ ^22^, ^23^ ^]^ Recent studies have shown that the fluorination of the phenylethylammonium spacer enhances the structural robustness of the crystal all the way to cryogenic temperatures^[^ ^24^ ^]^ and improves its resistance to ambient moisture and polar solvents,^[^ ^25^ ^]^ allowing for the reliable fabrication of nanoscale devices via standard top‐down lithography in ambient conditions,^[^ ^22^ ^]^ something rarely achievable with unmodified 2D perovskites. Owing to this unique stability, 2D F‐PEAI has already underpinned the development of multi‐functional healthcare and environmental sensing technologies^[^ ^23^ ^]^ demonstrating the potential of this emerging perovskite across a wide range of electronic and optoelectronic devices, extending its relevance far beyond the traditional perovskite focus on photovoltaics. Since trap states play a critical role in exciton dissociation and internal gain mechanisms across a broad range of materials—including perovskites and 2D transition metal dichalcogenides—the characterisation of defect states and their dynamics in operational 2D F‐PEAI devices is directly relevant to guiding the design and optimization of other layered perovskite materials and their device applications. For this study, transistors and photodetectors were fabricated from 2D F‐PEAI grown using previously demonstrated room‐temperature antisolvent vapor‐assisted crystallization, which led to high‐quality F‐PEAI single crystals, as evidenced by synchrotron X‐ray diffraction characterisation.^[^ ^26^ ^]^ In this system, low‐energy electronic excitations are confined to the layer of the inorganic [*PbI*_4_]^2 −^ anions, while the organic alkylammonium spacer functions as an insulating barrier leading to the confinement of charge carriers within the plane of the inorganic layer,^[^ ^27^, ^28^, ^29^, ^30^ ^]^ see **Figure** **1**a,b. Previous studies^[^ ^26^ ^]^ have shown that F‐PEAI has an energy bandgap of 2.61 eV (≈475 nm), and strongly bound excitons at room temperature dominating the photoluminescence (PL) spectrum with a sub‐gap energy emission peak at ≈523 nm, see Figure 1c and Section S0 (Supporting Information) for additional temperature dependent PL data. FETs and phototransistors were fabricated by laminating mechanically exfoliated flakes of F‐PEAI onto prepatterned Au contacts, with 3µm source‐drain channel length, on a doped Si/SiO_2_ substrate, which serves as a global back gate^[^ ^23^, ^26^ ^]^ (see Figure 1d), see Experimental Section and Section S0 (Supporting Information).

## 3 Electrical Transport Mechanisms in 2D F‐PEAI FETs

**Figure 2:**
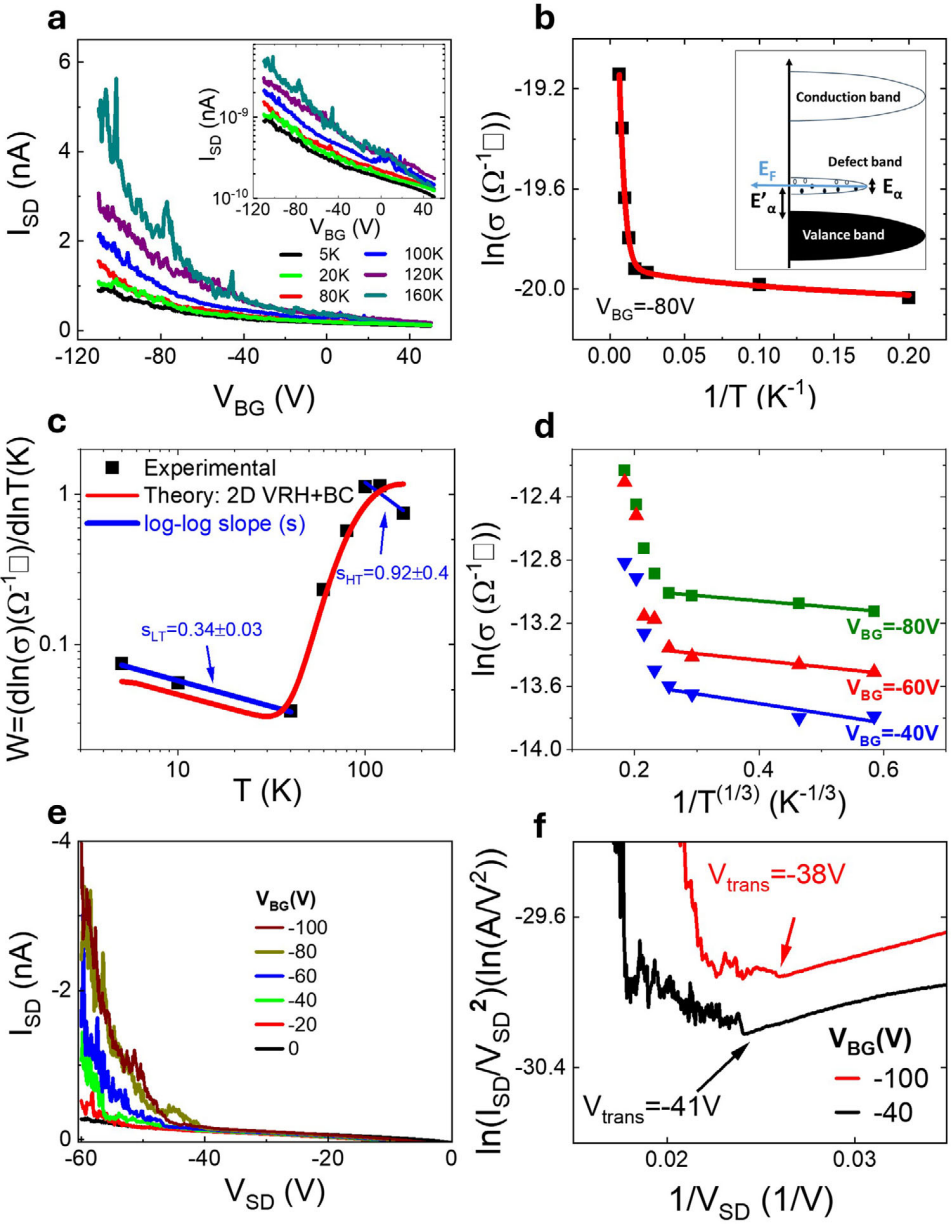
Temperature dependence of 2D F‐PEAI FETs transfer characteristic, Arrehnius plot of σ, analysis of reduced activation energy, 2D VRH scaling, FET output characteristics and Fowler‐Nordheim plots. a) Plot of the measured transfer characteristics for a 2D F‐PEAI FET for 20 K⩽*T* ⩽160 K, in linear and logarithmic scale main panel and inset, respectively. b) Arrhenius plot of the logarithm of the conductivity as a function of 1/*T*. The square points are experimental data, while the red line is the best fit with the multi‐channel conductivity, see main text. c) Logarithmic plot of the reduced activation energy, *W*, as a function of temperature. The red line is a plot of the multi‐channel conductivity using the parameters obtained in the best fit shown in panel (b). d) 2D VRH scaling plots for three different values of *V*_*BG*_. e,f) Plot of the output characteristics of the 2D F‐PEAI FET at 4.2 K and corresponding Fowler–Nordheim plot, respectively.

**Figure** **2**a shows measurements in dark conditions of the gate‐dependence of the source‐drain current (*I*_*SD*_) for fixed source‐drain bias *V*_*SD*_ = −45 V for a representative 2D F‐PEAI FET, see Supporting Information for data on additional devices. It is apparent that unipolar hole transport is observed across the temperature range 20 K⩽*T* ⩽ 250 K, consistent with the presence of a known asymmetric contact barrier for electrons and holes,^[^ ^22^ ^]^ see Section S1 (Supporting Information) for data >160 K. At the same time, the levels of source‐drain current measured in 2D F‐PEAI are of similar magnitude to those reported in other 2D FETs to include transition metal dichalcogneides and other 2D perovskites.^[^ ^31^, ^32^, ^33^ ^]^ A monotonic increase of *I*_*SD*_ is observed upon raising the temperature up to ≈160 K. For *T* > 160 K the *I*_*SD*_ gradually becomes temperature independent and finally also gate independent at 250 K. These observations suggest that at sufficiently high temperature, the back‐gate generated electric field drives the accumulation of mobile ions at the perovskite/gate dielectric interface, ultimately resulting in the complete screening of the back gate electric field lines. Indeed, a Nernst–Einstein plot analysis confirms a transition temperature and activation energy of the ionic movement of ≈120 K and *E*_α_ = 28.6meV, respectively (see Section S3, Supporting Information). Conversely, at cryogenic temperatures, the ion migration is suppressed and the gate modulation of the FET channel is observed, as recently reported by other authors.^[^ ^33^, ^34^, ^35^, ^36^ ^]^ Finally, detailed temperature‐dependent photoluminescence (PL) measurements from 6 K to 240 K (Figure S2, Supporting Information) reveal a single, well‐defined emission peak across the entire temperature range. The peak exhibits the expected monotonic broadening with increasing temperature, consistent with enhanced exciton–phonon interactions, and shows no anomalies indicative of a structural phase transition. In particular, no discontinuities or peak shifts are observed, unlike the abrupt PL changes reported at phase transitions in related materials^[^ ^34^ ^]^ (see Section S0, Supporting Information).

To understand the nature of the electrical transport in 2D F‐PEAIs close to the band gap edge and at sub‐gap energies, we analyse the temperature (*T*) dependence of the logarithm of the conductivity (σ) as a function of 1/*T* at T < 120K where the ionic contribution is negligible for fixed *V*_*BG*_ = −80 V and *V*_*SD*_ = −45 V, see Figure 2b. A crossover between two distinct transport regimes is observed at *T* ≈ 60 K, corresponding to an energy scale of ≈5 meV, which is two orders of magnitude smaller than the 0.3 eV Schottky barrier at the Au/F‐PEAI interface.^[^ ^22^ ^]^ In addition, the Fowler–Nordheim plot at T = 4.2K and T = 100K for the same back gate voltage (*V*_*BG*_ = −80*V*) shows a clear linear regime at high bias and a transition from direct to Fowler–Nordheim tunneling at *V*_*trans*_ = −39*V* (see Figure S5, Supporting Information). This suggests that for |*V*_*SD*_| > 39*V* charge carriers can easily traverse the sample due to the narrowing of the potential barrier.^[^ ^37^ ^]^ Thus, the electrical properties of the F‐PEAI channel rather than the perovskite/metal interface dominates the temperature dependence of the conductivity. At sub‐gap energies and in the presence of defect states, various hopping mechanisms can aid the flow of electrical current.^[^ ^38^ ^]^ These mechanisms generally result in thermally activated conductivity with a characteristic activation energy (*E*_*a*_) smaller than the energy gap of the semiconductor^[^ ^39^ ^]^ and a unique temperature dependence^[^ ^38^ ^]^ quantitatively described as $\sigma =\sigma_{0}e^{-{\left(E_{a}/k_{B}T\right)}^{\alpha }}$, where *k*_*B*_ is the Boltzman constant, σ_0_ is the conductivity in the limit *T* → ∞, and α is an exponent that provides information on the underlying conduction mechanism and the dimensionality of the system. For example, variable range hopping (VRH) is characterized by α = 1/(*d* + 1) where *d* is the dimensionality of the system, resulting in α = 1/3 for a 2D system as previously observed in other 2D systems such as fluorinated graphene^[^ ^40^ ^]^ and transition metal dichalcogenides.^[^ ^31^, ^41^ ^]^ On the other hand, α = 1 indicates a thermally activated transport mechanism, either through nearest‐neighbor hopping (NNH)^[^ ^42^ ^]^ or thermally activated band conduction (BC).^[^ ^43^ ^]^ BC typically exhibits a larger activation energy than NNH,^[^ ^44^, ^45^ ^]^ as it involves inter‐band activation of carriers from the defect band to the conduction band, whereas NNH only involves hopping of charges within the defect band, see inset in Figure 2b. Crucially, the microscopic mechanism underpinning the conductivity of a semiconductor can vary as a function of temperature. To this end, the analyses of the so‐called reduced activation energy, *W* = *d*(*ln*σ)/*d*(*lnT*), as a function of temperature is an established method to identify such transitions. A log–log scale plot of *W* directly provides the value of α = −*d*(ln *W*)/*d*(ln *T*), and its derivative with respect to the temperature (*dW*/*dT*) can be used to distinguish transitions from VRH to NNH or to BC occurring in intermediate temperature ranges.^[^ ^45^ ^]^

Figure 2c shows a log‐log plot of *W* = *d*(*ln*σ)/*d*(*lnT*) *vs* *T*. Three distinct regions, each characterized by a unique functional dependence of *W*(*T*), are clearly visible. For *T* < 40 K, a fit of the experimental data yields α = 0.34 ± 0.03, indicating that 2D VRH is the dominant microscopic mechanism for the electrical conduction. In contrast, for *T* > 100 K the exponent α approaches a value of 1, marking the onset of thermally activated transport. In the intermediate temperature range (40 K < *T* <100 K), the system transitions into a mixed transport regime. To quantitatively describe the conduction across the entire temperature range, a phenomenological model is adopted, incorporating the multi‐channel conductivity expressed as 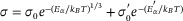. The red line in Figure 2b,c shows the best fit of the experimental data with values *E*_α_/*k*_*B*_ = 0.3 K, 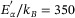 K, σ_0_ = 2.4 × 10^−9^Ω^−1^□ and 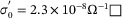. The activation energy of the low‐temperature 2D VRH hopping regime is much smaller than the energy gap of F‐PEAI (2.61 eV), indicating that electrical transport occurs within a narrow defect band. This low‐temperature hopping mechanism persists across the range 40 V⩽*V*_*BG*_ ⩽ 80 V, see Figure 2d. At higher temperatures, a new conduction mechanism emerges, consistent with thermally activated transport characterized by a much larger activation energy than that of 2D VRH. This indicates the activation of charge carriers from the defect band into the valence band. The transition is evidenced by the large positive slope of *dW*/*dT*, consistent with a transition from 2D VRH to band conduction (BC) rather than nearest‐neighbor hopping (NNH).^[^ ^45^ ^]^ Finally, BC dominates the electronic transport for *T* = 152 K (see Figure S7, Supporting Information). Additionally, temperature dependent tranfer characteristic measurements of more samples confirm the same mixed thermal activated 2D VRH and band conduction at low temperatures (see Figure S9, Supporting Information).

## 4 Threshold Voltage Transient Spectroscopy

A close analysis of the 2D F‐PEAI FET transfer characteristics reveals large hysteresis and shift of the threshold voltage Δ*V*_*th*_ ≃ 36 V as well as a considerable subthreshold voltage swing *S*_4.2*K*_ = *dV*_*BG*_/*d*log *I*_*D*_ = 200 V/dec, see Section S4 (Supporting Information). These are strong indicators of the presence of sub‐gap charge trapping states, likely due to iodine vacancy.^[^ ^22^ ^]^ Extracting information on these trap states and their role on the charge carrier dynamics directly from the transfer characteristics normally requires the use of contacts with negligible barriers. The sublinear trend and high transition voltage (*V*_*trans*_) observed in the output transfer characteristics and corresponding Fowler‐Nordheim plots^[^ ^33^ ^]^ for 2D F‐PEAI FET indicate the presence of a large barrier at the F‐PEAI/Au interface,^[^ ^22^, ^33^ ^]^ see Figure 2e,f and Section S2 (Supporting Information). Recent threshold voltage transient spectroscopy studies in 2D transition metal dichalcogenides FETs showed that, contrary to other techniques, TVTS is contact resistance tolerant, granting direct insight on the energy scales of defect states and trapping dynamics at a fixed temperature without the need to acquire an Arrhenius plot.^[^ ^19^ ^]^ Presently, the potential of TVTS has not yet been explored in fully processed 2D perovskite devices.

**Figure 3:**
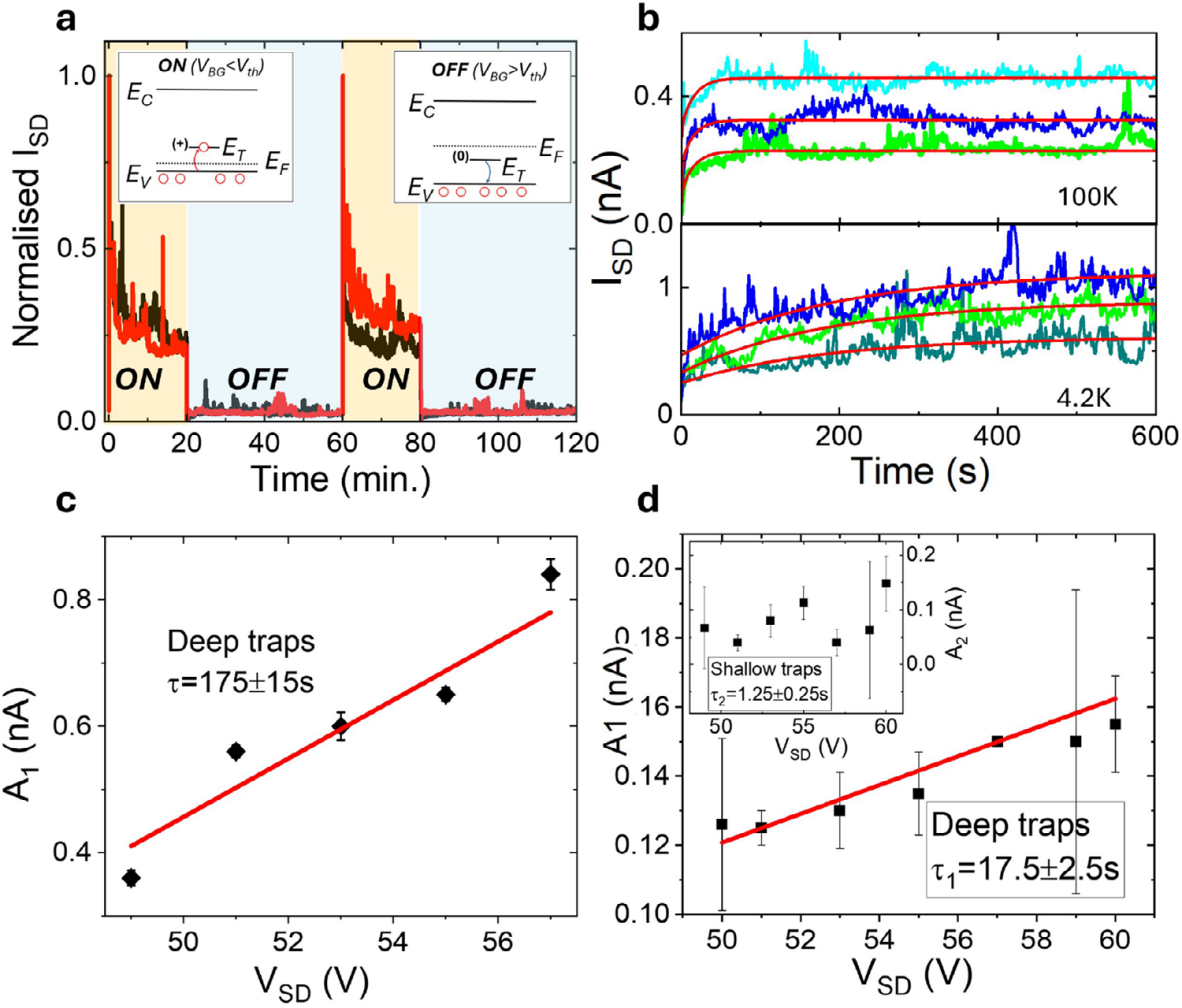
Emission and capture processes, pulsed gate transient measurements, time‐resolved emission current, bias dependence of the emission coefficient. a) Plot of the time resolved normalised *I*_*SD*_ during the capture (ON) and emission (OFF) cycles triggered by *V*_*BG*_ pulses (–60 V, ON, and 0 V, OFF) at 4.2 K (black) and 100 K (red), and for a fixed *V*_*SD*_ = 55 V. Insets: energy band diagrams for the capture (ON) and emission (OFF) processes, respectively. *E*_*C*_, *E*_*V*_, *E*_*F*_, *E*_*T*1_, and *E*_*T*2_ are the conduction‐band minimum, the valance‐band maximum, the Fermi energy, the shallow midgap state, and deep midgap state energy, respectively. b) Plots of time‐resolved *I*_*SD*_ in the emission state recorded at *V*_*BG*_ = 0 V for *V*_*SD*_ = 49 V (olive), 51 V (green) and 55 V (blue) at 4.2 K (bottom) and for *V*_*SD*_ = 51  (green), 55 V (blue) and 57 V (cyan) at 100 K (top), see Section S7 (Supporting Information) for additional data. The red curves are an exponential fit, see the main text. c,d) plots of the bias dependence of the pre‐exponential emission coefficient at 4.2 and 100 K, respectively. The red line represents the best linear fit.

The schematics shown in **Figure** **3**a illustrate the processes of charge trapping and de‐trapping in the energy band diagrams, emphasizing the role of sub‐gap states. In the ON‐state, the Fermi level lies below the trap energy levels, rendering these states unoccupied by electrons (effectively occupied by holes). This is referred to as the capture mode, where positively charged traps cause a shift in the threshold voltage *V*_*th*_ toward more negative values. Conversely, in the OFF‐state, the Fermi level moves above the traps energy levels, enabling the traps to be filled by electrons and emit holes into the valence band. This process, known as the emission mode, makes the defects charge neutral. The associated charge dynamics of these charges in the trap states forms the foundation of TVTS, which uses voltage pulses applied to the gate electrode to drive the system between an “open” (more negative) to a “close” (more positive) value. Figure 3a shows the time‐dependent drain current (*I*_*SD*_) measured over extended periods of time while repeatedly pulsing the gate voltage between *V*_*BG*_ = −60 V (to open the channel) and *V*_*BG*_ = 0 V (to close it). Upon applying the negative pulse to open the channel, *I*_*SD*_ initially increases rapidly due to the sudden activation of the channel. Subsequently, *I*_*SD*_ slowly decays as *V*_*th*_ shifts toward more negative values. When *V*_*BG*_ = 0 V, the device transitions to the off‐state, with the traps entering the emission mode. This results in a sudden drop in *I*_*SD*_ as the channel is turned off. Over time, the saturation current (*I*_0, *sat*_) is gradually restored as *V*_*th*_ shifts back to more positive values, see Figure 3b–d. These observations highlight the interplay between gate voltage and charge trapping/detrapping dynamics in 2D F‐PEAI FETs.

Time evolution of source‐drain current (*I*_*SD*_) on a 2D channel reflects the decay of the occupied traps due to the *V*_*th*_ shift of the 2D channel and the exponential rise of *I*_*SD*_ that can be calculated using the dynamic equation of the occupied trap states. If we assume a p‐type channel and Shokley Read Hall statistics, the time evolution of the density of the occupied traps after the pulse is equal to *n*_*t*_(*t*) = *n*_*t*_(∞) + (*N*_*t*_ − *n*_*t*_(∞))*e* ^−(*t*/τ)^ where *n*_*t*_(∞) = *N*_*t*_(*c*_*p*_ *p*/(*c*_*p*_ *p* + *e*_*p*_)) is the number of the occupied traps at the low injection steady state (OFF‐mode of the pulse), *N*_*T*_ the density of all the traps and τ = 1/(*c*_*p*_ *p* + *e*_*p*_), while *p* is the concentration of holes at the low injection (OFF‐mode) and *c*_*p*_, *e*_*p*_ their capture and emission coefficients (see Section S7, Supporting Information). When the low injection level of the pulse actually corresponds to a depleted channel (*V*_*BG*_ = 0*V*), *p* is zero and *n*(*t*) is simplified to *n*_*t*_ ∼ *N*_*t*_ *e* ^−(*t*/τ)^ and τ = 1/*e*_*p*_. This case is equivalent to the decay of the filled traps after a light pulse as only the thermal emission of carriers from the traps governs the detrapping process.^[^ ^13^ ^]^ Besides, if the channel during the OFF mode is not fully depleted, and it presents a non‐zero concentration of carriers (*V*_*BG*_ ≠ 0*V* → *p* ≠ 0) capture process affects the detrapping dynamics, drastically. Capture rate and retrapping of carriers at the trapping centers (≈*c*_*p*_ *p*) are directly proportional to the concentration of the carriers into the channel and the retrapping effect can be strongly enhanced after the constant injection of carriers into the channel. Thus, TVTS enables an excquisite control of the trapping dynamics, and it also probes the detrapping dynamics not only when pure thermal assisted emission of trapped carriers is the dominant detrapping mechanism, but also when strong retrapping occurs due to enhanced re‐capture of carrier by the traps following the back gate field injection of carriers into the channel. Indeed, strong retrapping is found to play a role in 2D perovskites, as shown by the characteristic fluctuations in the log–log decay of discrete traps^[^ ^13^ ^]^ and time evolution of *I*_*SD*_ (see Section S7, Supporting Information).

The transient channel current in the linear regime for the transistor can be calculated using the well known equation,^[^ ^19^, ^46^ ^]^ *I*_*SD*_ = (*W*µ_*p*_ *C*_*ox*_/*L*)(*V*_*th*_ − *V*_*BG*_)*V*_*SD*_:

(1) $$I_{SD}\left(t\right)=I_{0,sat}-\frac{qW\mu_{p}N_{T}V_{SD}}{L}e^{\left(-t/\tau \right)}$$

where *q* is the elementary charge, *W* and *L* are the width and the length of the channel, µ the mobility of the majority carriers. At the same time, as shown in ref. [19] the emission of charges from a depletion region of a conventional semiconductor, with negligible *V*_*th*_ shift:

(2) $$I_{SD}\left(t\right)=I_{0,sat}+\frac{qN_{T}LW}{\tau }e^{\left(-t/\tau \right)}$$

Hence, the time evolution of the prefactor of the exponential can be used to identify the presence of traps in the 2D channel and the dominance of the threshold voltage change. Indeed, Figure 3b presents a rise exponential behavior of channel current (*I*(*t*) ≈ *I*_*sat*_ − *I*_0_ *e* ^−(*t*/τ)^) rather than a decay exponential behavior (*I*(*t*) ≈ *I*_*sat*_ + *I*_0_ *e* ^−(*t*/τ)^) after the OFF‐back gate pulse confirming even further the 2D nature of the perovskite layers and the validity of TVTS on the 2D F‐PEAI FETs, as the entire channel is uniformly affected by the trapped charges, and *V*_*th*_ follows the decay of the occupied traps. In addition, this model also predicts that trap states with different dynamics, such as deep and shallow traps, result in a double exponential dependence of *I*_*SD*_.^[^ ^19^ ^]^ Using TVTS, we can quantitatively describe the measured transient emission current in 2D F‐PEAI with the single rise exponential $I\left(t\right)=I_{0}-A_{1}e^{\left(-t/\tau_{1}\right)}$ at 4.2 K, whereas the data at 100 K are best fit using the double rise exponential $I\left(t\right)=I_{0}-A_{1}e^{\left(-t/\tau_{1}\right)}-A_{2}e^{\left(-t/\tau_{2}\right)}$ where *A*_1_ and *A*_2_ are the so‐called emission coefficients, see Figure 3b and Section S7 (Supporting Information) for the fitting method. Finally, the plots in Figure 3c,d show the *V*_*SD*_ dependence of the emission coefficient obtained, resulting from the fit of the experimental data conducted without imposing any constraints on the values of the pre‐exponential factor.

**Figure 4:**
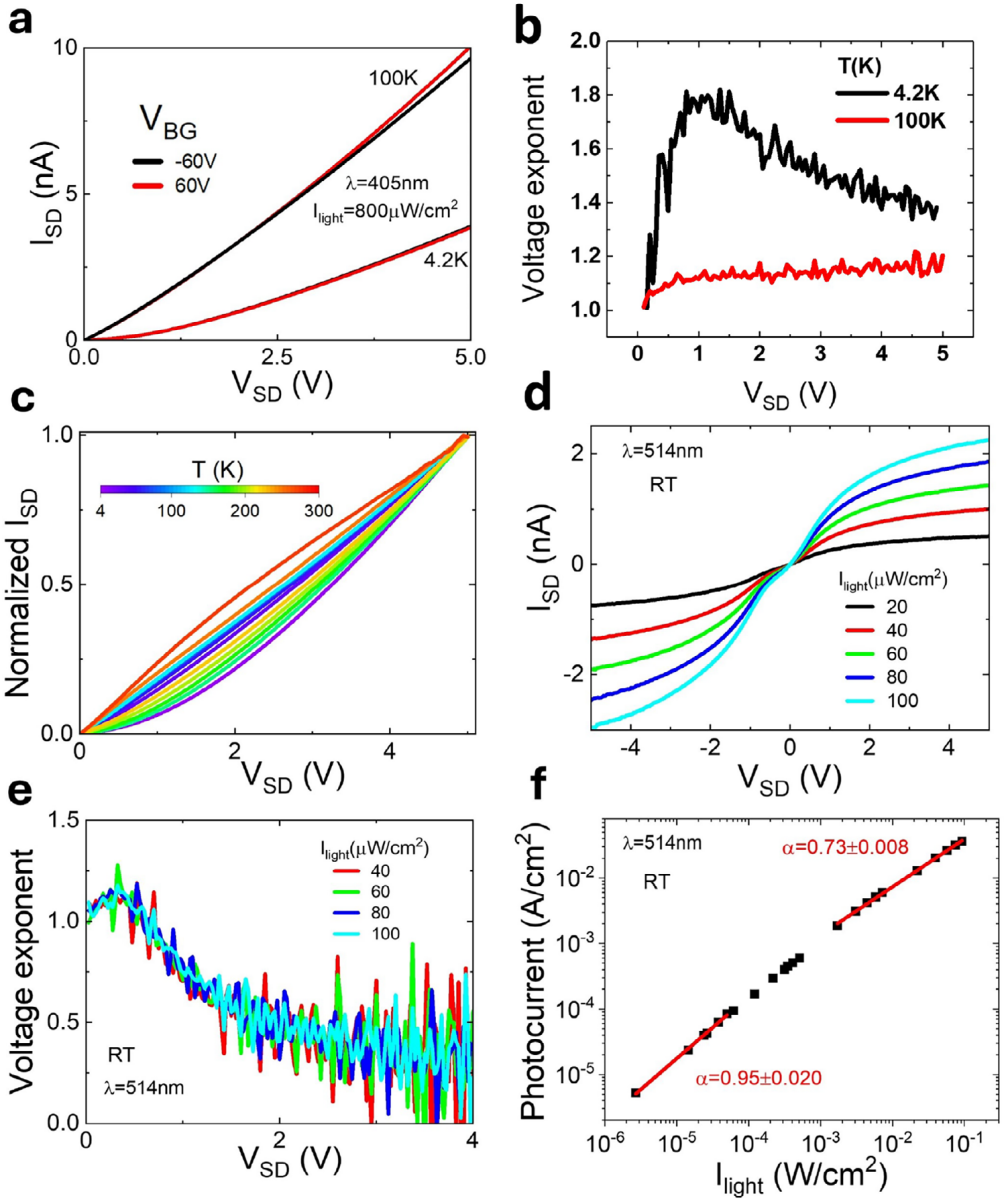
Light irradiance and temperature dependence of the 2D F‐PEAI photodetector current bias characteristics.(a) Plot of the measured *I*_*SD*_ vs. *V*_*SD*_ illuminating the whole photoactive area of the photodetector ( *A*_*act*_ ≈ 19000 µm^2^) with UV light λ = 405 nm and irradiance *I*_*light*_ = 800 µW cm^−^^2^, and for fixed values of *V*_*BG*_. (b) Log‐log slope (voltage exponent) of *I*_*SD*_ vs. *V*_*SD*_ for *V*_*BG*_ = −60*V* values shown in (a) and at 4.2 and 100 K, respectively. (c) Plot of the normalized *I*_*SD*_ *vs* *V*_*SD*_ measured at various temperatures from 4.2 to 297 K while illuminating with λ = 405 nm (*I*_*light*_ = 1 mW cm^−^^2^). (d) Plot of the room temperature bias dependence of the *I*_*SD*_ measured illuminating the device with λ = 514 nm for various levels of irradiance as specified in the legend. (e) Plot of the log–log *I*_*SD*_ slope for the data in (d). (f) Plot of the irradiance dependence of the photocurrent measured at constant bias, *V*_*SD*_ = 5 V, and uniform illumination of the whole photo‐active device area, 1300 µm^2^, with monochromatic light, λ = 514 nm of varying irradiance.

The thermal activation of the detrapping effect is confirmed by the temperature dependence of its characteristic time, which decreases from τ_1_ = 165 ± 15 s at 4.2 K to τ_1_ = 20 ± 2 s and τ_2_ = 1.25 ± 0.25 s at 100 K. In addition, a plot of the *V*_*SD*_ dependence of the emission coefficient *A*_1_ at 4.2 K reveals a clear linear dependence consistent with the presence of uniformly distributed deep trap states throughout the 2D F‐PEAI with sub‐gap energies following the SRH statistics, which entails exponential time dependent trap dynamics,^[^ ^19^ ^]^ see Figure 3c. A double exponential is required to correctly fit the experimental data at 100 K. The first pre‐exponential factor *A*_1_ shows a linear dependence on *V*_*SD*_ in the range >51 V when the threshold voltage transient effect exceeds the emission current,^[^ ^19^ ^]^ see Figure 3d. At the same time, the second pre‐exponential factor *A*_2_ is found to be independent of *V*_*SD*_ indicating the presence of shallow traps, see inset Figure 3d and Section S7 (Supporting Information) for similar data on additional devices. Indeed, in this temperature range, two distinctive de‐trapping times τ_1_ = 20 ± 2.5s and τ_2_ = 1.25±0.25s are observed, corresponding to deep and shallow traps, respectively. This finding is consistent with the observation that at low temperature (*T* ⩽40 K) 2D VRH of carriers through the deep defect states is the microscopic mechanism aiding electrical conduction. However, for temperatures 40 K⩽*T* ⩽152 K, the presence of shallow traps plays a progressively more prominent role as the system transitions to thermally activated transport, and the deep trap population decreases from 9.3 ± 0.7 × 10^12^ cm^−2^ at 4.2K to 3.1 ± 0.3 × 10^11^ cm^−2^ at 100K, see Experimental Section. To further validate these findings on the trap dynamics, back‐gate pulsed measurements were performed at higher *V*_*SD*_ values at 100 K (see Section S6, Supporting Information). Additionally, the coexistence of deep and shallow traps was confirmed across multiple samples, see Figure S7 (Supporting Information).

## 5 Impact of Trap States on Photoconductivity

To unveil the role of trap states in the photoconductivity of 2D F‐PEAI, we studied the temperature and bias dependence of the photocurrent generated upon illuminating the whole photoactive area of planar devices with a UV light (λ = 405 nm and irradiance 800 µW cm^−^^2^), see **Figure** **4**a and Supporting Information S8 and S9. In all cases, a large *I*_*SD*_ is measured, which is >1000 times larger than the dark current (*I*_*dark*_ < 5 × 10^−12^ A for *V*_*SD*_ < 10 V and *V*_*BG*_ = 0 V, see Figure 2a). Upon lowering the temperature from 100 to 4.2 K, the values of *I*_*SD*_ decrease and its dependence on *V*_*BG*_ diminishes . The voltage dependence of the photocurrent slope, *s* = *d*(*log*(*I*_*ph*_))/*d*(*log*(*V*_*SD*_)), plotted as a function of *V*_*SD*_ reveals a superlinear behavior at 4.2 K, with a local maximum of the voltage exponent reaching up to 1.8, see Figure 4b. In contrast, at 100 K the voltage exponent increases monotonically as a function of *V*_*SD*_, approaching a nearly constant value of 1.2 for *V*_*SD*_>1 V. A plot of *I*_*SD*_(*V*_*SD*_) normalised to its measured values at *V*_*SD*_ = 5 V and for a larger set of temperatures reveals a significant change in the functional dependence of *I*_*SD*_(*V*_*SD*_) from superlinear at low temperatures to sublinear at room temperature, see Figure 4c and Supporting Information S8 and S9. The sublinear functional dependence persists for other wavelengths with photon energy above the 2D F‐PEAI exciton energy,^[^ ^22^ ^]^ such as 514nm, and for various levels of light irradiance (20–100 $\mu Wcm^{-2}$), see Figure 4d. Consistently, the slope of the log–log plot of *I*_*SD*_ *vs* *V*_*SD*_ approaches zero as the source‐drain current saturates, see Figure 4e.

Blocking metal contacts are known to drive a saturating *I*_*SD*_ as a function of *V*_*SD*_ upon illumination, while sub‐gap trap states increase the value of *V*_*SD*_ from which such a saturation is achieved.^[^ ^47^, ^48^ ^]^ In this case, the photogenerated carriers are extracted from the optical active area with a linear^[^ ^47^ ^]^ or superlinear^[^ ^48^ ^]^ depending on sub‐gap traps. Upon increasing the bias, the mean drift length of the carriers (*L*′) increases, and when this becomes equal to the channel length, the photogenerated carriers are extracted from the channel before they can recombine. The photocurrent is $I_{ph}=I_{SD}-I_{dark}=eG\mu \tau V_{SD}^{1/2}$, where *e* is the elementary charge and *G* the photo‐generation rate of carriers, that is proportional to the irradiance. At sufficiently large values of *V*_*SD*_ the photocurrent saturates due to the limited injection of carriers.^[^ ^47^ ^]^ In 2D F‐PEAI, this scenario is further influenced by sub‐gap defect states, which increase the dwell time of one carrier type resulting in unbalanced transport. Sub‐gap states, combined with blocking contacts, lead to the accumulation of slow carriers near one contact, which in turn significantly modifies the externally applied electric field at high irradiance.^[^ ^47^, ^49^ ^]^ This carrier accumulation at the F‐PEAI/contact interface limits the extraction of the photocurrent, resulting in space‐charge‐limited photocurrent (SCLP). This is described by $I_{SCLP}={\left(\frac{9\mu_{h}\varepsilon_{0}\varepsilon }{8e}\right)}^{1/4}G^{3/4}V^{1/2}$, where µ_*h*_ is the mobility of the slow carrier and ϵ_0_, ϵ is the dielectric constant of the vacuum and the material, respectively. The unique functional dependence of *I*_*ph*_ and *I*_*SCLP*_ on *G* provide a clear way to identify the main microscopic mechanism at play.^[^ ^47^, ^49^ ^]^ Figure 4f shows a log‐log plot of the measured photocurrent as a function of the irradiance for the same device, see Supporting Information for data on additional samples. A best fit of the photocurrent at high irradiance reveals a slope value of ≈0.75, consistent with SCLP. In contrast, at low irradiance, the slope approaches a value of 1 as expected for blocking contacts when the low photo‐generation rate is insufficient for the system to enter the SCLP regime. Hence, the overall optoelectronic response of the Au/2D F‐PEAI/Au system is governed by the interplay of metal contacts and shallow trap states, which induce unbalanced transport of photogenerated carriers.

**Figure 5:**
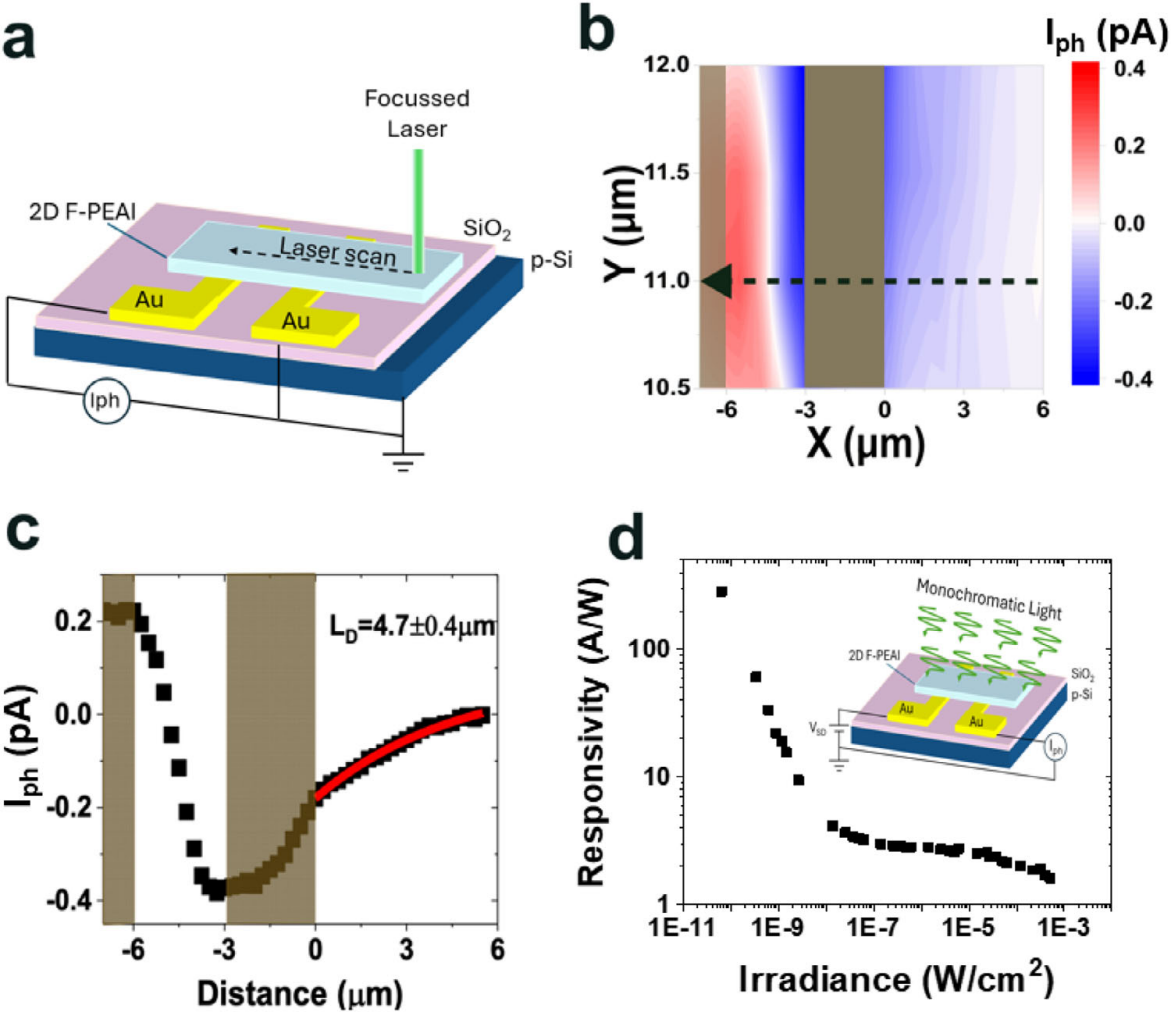
Characterisation of the photoexcited charge carrier diffusion and figures of merit of the photodetector. Panel a) shows a schematic view of a 2D F‐PEAI photodetector with circuit diagram used for the characterisation of the photoexcited carrier diffusion by illuminating with a focused laser beam (wavelength 514 nm, laser spot diameter *d*_*laser*_ = 0.5 µm, power *P*_*laser*_ = 10 nW and scanning step 0.25 μm). b) Color plot of the measured spatially resolved photocurrent acquired using the configuration shown in (a) with *V*_*SD*_ = 0 V. The grey shaded areas correspond to the source and drain Au electrodes. c) shows a line cut from the measurements in panel (b) in the direction of the dashed arrow line. The red line is the exponential fit with the diffusion length *L*_*D*_ of photoexcited carriers as a fitting parameter. d) Plot of the measured photoresponsivity and EQE as a function of the irradiance acquired by illuminating the whole photoactive surface area *A*_*active*_ = 1300 µm^2^ (λ = 514 nm) and applying a constant *V*_*SD*_ = −5 V following the circuit configuration shown in the inset.

Earlier studies on the photo‐excited carrier diffusion length (*L*_*D*_) in other in semiconductors have highlighted the possible beneficial role of shallow traps and retrapping effect in extending carrier lifetimes and enhancing *L*_*D*_.^[^ ^9^, ^10^, ^11^ ^]^ Hence, to characterize *L*_*D*_ in 2D F‐PEAI, we measure the spatial distribution of the photocurrent at zero bias by rastering a focused 514 nm continuous wave laser with a beam diameter 0.5 µm and fixed laser power 10 nW over the photodetector,^[^ ^48^, ^50^ ^]^ see **Figure** **5**a–c. The resulting line scans exhibit a minimum (maximum) value of photocurrent near the source (drain) electrode, consistent with the presence of Schottky contacts.^[^ ^50^ ^]^ At the metal/semiconductor interface, downward band bending facilitates the electron extraction while blocking holes, leading to a reversed photocurrent upon illuminating the 2D F‐PEAI region covering the source and drain metal contacts.^[^ ^50^ ^]^ At zero bias the Schottky barrier at the metal contacts also filters the diffusion current of the minority carriers.^[^ ^50^ ^]^ When the laser spot moves outside the source‐drain 2D F‐PEAI region, the photoexcited carriers diffuse into the depleted regions producing a photocurrent signal that decays exponentially with a characteristic diffusion length *L*_*D*_.^[^ ^50^, ^51^ ^]^ The plot in Figure 5c shows the exponential decay of the photocurrent (*I*_*ph*_ ∼ *exp*(− *x*/*L*_*D*_)) in the regions outside the metal contacts for the line cut highlighted in Figure 5b. The extracted *L*_*D*_ is 4.7 ± 0.4 µm, significantly exceeds the source‐drain channel length. Conducting a similar exponential fit for each of the raster laser lines forming the map of Figure 5b yields an overall average value of the diffusion length of $\bar{L_{D}}=4.5\pm 0.5\mu m$. This macroscopic diffusion length is among the best values reported in the presence of shallow traps.^[^ ^9^, ^10^, ^11^, ^50^ ^]^

Finally we evaluate the role of traps on the photoresponsivity and gain mechanism in 2D F‐PEAI photodetectors.^[^ ^22^ ^]^ Figure 5d shows a plot of the measured photoresponsivity, *R* = *I*_*ph*_/*I*_*light*_ *vs* light irradiance in the range *I*_*light*_ = 5 × 10^−11^ − 4 × 10^−4^ W cm^−^^2^ for a fixed *V*_*SD*_ = −5 V and by illuminating the whole device photoactive area with monochromatic light λ = 514 nm. The corresponding irradiance dependence of the external quantum efficiency, *EQE* = (*hc*/*e*λ)*R*, is shown in the Figure S8 (Supporting Information). Both *R* and *EQE* increase monotonically upon reducing the irradiance, reaching values of *R* = 120A W^−1^ and *EQE* ≈ 260 at 5 × 10^−11^ W cm^−^^2^. An EQE > 100% is indicative of an internal gain mechanism, such as photoconductive gain, where a single photogenerated carrier can contribute to multiple charge collection events. Recent studies on 2D materials have demonstrated that defect or trap states can facilitate this process by transiently capturing one carrier type (typically the slower one), thereby extending its lifetime and allowing the opposite carrier to recirculate under an applied bias, effectively amplifying the photocurrent.^[^ ^13^, ^52^ ^]^ While Schottky barriers at the metal/semiconductor interface are generally expected to impede charge carrier extraction, recent studies on 3D perovskites^[^ ^53^, ^54^ ^]^ and 2D semiconductors^[^ ^55^, ^56^ ^]^ have demonstrated that interfacial trap filling by photoexcited carriers can reduce the barrier height. This mechanism enhances the injection of fast photoexcited carriers. Additionally, the accumulation of slow carriers near the blocking metal contact can induce image‐force effects, contributing to lowering the interface barrier.^[^ ^57^ ^]^ These mechanisms enable 2D F‐PEAI photodetectors to detect ultra‐low light signals owing to a detectivety *D* ∼ 1 × 10^17^ Jones at irradiance *I*_*light*_ = 5 × 10^−11^ W cm^−^^2^ and a noise equivalent power of ≃ 10^−17^ A/$\sqrt{Hz}$, see Section S11 (Supporting Information).

## 6 Conclusion

In conclusion, we demonstrate a robust and yet simple method for the characterisation defect states and their dynamics in fully processed multifunctional optoelectronic devices based on 2D perovskites under operating conditions. Utilizing transient voltage spectroscopy, we elucidate the dynamics, types (i.e. deep and shallow defects), and the number density of sub‐gap trap states that critically affect the performance of 2D F‐PEAI single‐crystal‐based field‐effect transistors and photodetectors. These trap states manifest through threshold voltage shifts, hysteresis effects, and temperature‐dependent charge trapping and detrapping dynamics. At cryogenic temperatures (up to 60 K), carrier transport is dominated by deep trapping effects, transitioning to band conduction at higher temperatures. At room temperature, space‐charge‐limited photoconduction emerges due to Schottky barriers at the metal contacts and trap‐assisted unbalanced transport of photoinduced carriers. Scanning photocurrent measurements of Au/2D F‐PEAI/Au structures reveal a long carrier diffusion length (on average 4.5 ± 0.5 µm), while trap states enable internal gain mechanisms boosting the external quantum efficiency at 2600% and overall sensitivity of the photodetectors. While numerous techniques have been developed for characterizing trap states in 3D semiconductors, only a limited subset is applicable to 2D systems, particularly when the thickness of the active layer is smaller than the Debye length. Among these, none allow for the characterisation of fully fabricated 2D transistors without requiring modifications that alter their normal operating conditions. In this work, we introduce a trap dynamics characterisation method compatible with fully processed devices, thus addressing a critical gap between trap states in unprocessed materials and their devices under operating conditions. This blind spot hinders the understanding of trap behavior in 2D perovskite devices under realistic operational scenarios and their exploitation for a wider range of multifunctional FET applications than photovoltaics. A future further development of this technique could enable the extraction of the sub‐gap density of states as a function of gate bias at a fixed temperature—provided the work function and electron affinity of the 2D perovskite are known.^[^ ^20^ ^]^ Collectively, our findings reveal a valuable method to link specific electric transport mechanisms (e.g. band transport vs nearest neighbour‐ and variable range‐hopping) to trap properties (density, type and dynamics) at the operating temperature of the devices, providing actionable parameters for engineers to tune device architectures for multifunctional transistors, diodes, memories, sensors, and beyond.

## 7 Experimental Section

### Fabrication of Contacts

Doped Si/SiO_2_ substrates were coated by layer of ≈400 nm of 450K A6 PMMA (spinning speed 2000 rpm), baked on hot plate at 180 °C for 3 min, exposed to a 100 keV e‐beam lithography for the patterning of the electrodes using a dose of 1000 µC cm^−^^2^, and developed for 1 min 30 s in a solution of IPA:MIBK (3:1 volume ratio). The metal deposition was achieved using e‐beam evaporation of Ti/Au (5/30 nm) followed by lift‐off in warm acetone (at 70 °C) for 1 h, see Supporting information S0. Interdigitated electrodes were employed to maximize the contact area within a compact footprint, thereby reducing contact resistance and enhancing carrier collection efficiency in the 2D perovskite devices, see Section S0 (Supporting Information).

### Synthesis of (F‐PEA)_2_PbI_4_

F‐PEAI single crystals were synthesized with the antisolvent vapor‐assisted crystallization method carried out at room temperature, following the procedures described in prior work.^[^ ^22^, ^23^ ^]^ Briefly, 267 mg 4‐Fluoro‐Phenethylammonium iodide and 230.5 mg PbI_2_ were dissolved in 1 mL of gamma‐butyrolactone and stirred at 70 °C for 30 min. A N_2_‐filled glovebox were used to prepare the precursors solutions. The synthesis of crystals took place by confining the perovskite solution (2 µL) between two glass slides cleaned by any surface organic contaminants. A small vial containing 2 mL of dichloromethane was placed on top of the glass slides, and the whole system was then enclosed in a Teflon vial, closed with a screw cap and left undisturbed for 12 h.

### Exfoliation and Transfer of (F‐PEA)_2_PbI_4_

Single crystals of 2D F‐PEAI were mechanically exfoliated following the procedure described in refs. [23, 26] using adhesive thermal‐release tape (Graphene Supermarket, SKU: GTT‐5P), see Section S0 (Supporting Information). Thin and uniform flakes were first identified under a white‐light optical microscope based on contrast and morphology. Suitable flakes—those exhibiting large lateral dimensions and uniform thickness—were aligned over a target substrate prepatterned with gold electrodes. The tape was then manually brought into contact with the substrate to allow adhesion of the flakes. To complete the transfer, the tape and substrate assembly was heated to 95 °C for 10 s, releasing the flakes onto the substrate. The visibility of the gold pads and electrodes through the semi‐transparent thermal‐release tape provides sufficient contrast for alignment.

### Temperature Dependent Measurements

The temperature dependence of the electrical and opto‐electronic properties of the devices was measured using a ICE‐Oxford cryogenic insert with a custom‐built sample socket housing a LED light source. This system offers accurate control over the sample temperature from 4.2 K to room temperature. Keithley 2400 source‐meter instruments were used to apply a constant voltage bias to the source of the 2D F‐PEAI devices. The current to the drain contact was measured connecting the drain to an Ithaco 1211 current amplifier, whose output was read out by a Keysight 34401A. The conductance (*G*) of the devices was calculated by dividing the measured drain current (*I*_*SD*_) by the value of the applied voltage bias (*V*_*SD*_), i.e. *G* = *I*_*SD*_/*V*_*SD*_. Finally, the electrical conductivity (σ) for the 2D F‐PEAIchannel system was given by σ = *GW*/*L* where *W* and *L* were the known 2D F‐PEAI channel width and length. A second Keithley 2400 source‐meter instrument was used to control the LED light.

### Photoluminescence, Photoactive Area and Photo‐Excited Carrier Diffusion

Photoluminescence, photocurrent maps for determining the photoactive area and photo‐excited carrier diffusion were acquired using a custom‐built optoelectronic characterisation system optimized to probe the photophysical properties of 2D materials.^[^ ^58^ ^]^ The system embeds a number of solid state laser sources (Coherent OBIS 375LX, 473LS, 514LX, and 561LS and Omicron LuxX 685, with powers ranging from 30 to 50 mW). Each laser was digitally modulated, and the power was adjusted using an analog signal. Custom‐built drop‐in‐filter systems were used to introduce commercial neutral density, polarisers, notch, and bandpass filters in the optical path of the lasers and the microscope. The spectrometer was a Princeton Instruments Acton SP2500, equipped with three dispersion gratings (1200 g mm^−1^ with 500 and 750 nm blaze, and 1800 g mm^−1^ with 500 nm blaze) and it was equipped with a Princeton Instruments PIXIS400‐eXcelon back‐illuminated, Peltier cooled, CCD camera. The optical path can be configured for Raman, photoluminesce, and transmission/reflection spectroscopy and laser light illumination for photocurrent maps simply by replacing or removing the appropriate filters. The sample stage was a Prior Scientific OptiScan ES111 with a ProScan III controller with a minimum step size of 100 nm, enabling the accurate control of focused laser light for photocurrent maps and photo‐excited carrier diffusion length. Calibrated power meters and fast photodetectors were used to measure the light intensity.

### Characterisation of Photoresponsivity and Photodetectivity

Spectrally resolved photo response measurements were acquired using a Xenon lamp and monochromator (*Newport* TLS300X) with light intensities adjusted using OD filters. All light source intensities were calibrated using a calibrated photo‐diode (Thorlabs S130CV). The noise spectral density was acquired using a Rohde & Schwarz RTO Oscilloscope with real time spectral capabilities and a Rohde & Schwarz FSU Spectrum Analyser.

### Traps Density

The traps number density was calculated from *N*_*traps*_ = *L*(*dA*_1_/*dV*_*SD*_)/(*qW*µ_*p*_)^[^ ^19^ ^]^ that it can be simplified in *N*_*traps*_ = *C*_*ox*_ *V*_*SD*_(*dA*_1_/*dV*_*SD*_)/(*q*(*dI*_*SD*_/*dV*_*BG*_)) after using the relation µ_*p*_ = *L*(*dI*_*SD*_/*dV*_*BG*_)/(*WC*_*ox*_ *V*_*SD*_),^[^ ^59^ ^]^ where *L*, *W* were the actual length and the width of the device, while *C*_*ox*_ = ϵ_0_ϵ_*r*_/*d* = 115µ*Fm* ^−2^ was the dielectric capacitance of the back gate oxide, with ϵ_0_ the vacuum permittivity, ϵ_*r*_ the relative permittivity of the oxide (*SiO*_2_), and *d* the thickness of the oxide (*d* ≃ 300*nm*). Hence, from the data shown in Figure 3c,d *dA*_1_/*dV*_*SD*_ was estimated to be 4.6 ± 1 × 10^−11^ A/V at 4.2K and 4.1 ± 0.3 × 10^−12^ A/V at 100 K. While, from the transfer characteristics of the device (see Figure 2a) and for *V*_*SD*_ = 45V, *dI*_*SD*_/*dV*_*BG*_ was calculated to be 1.6 ± 0.08 × 10^−11^ A/V at 4.2K and 4.2 ± 0.14 × 10^−11^ A/V at 100 K.

## Conflict of Interest

The authors declare no conflict of interest.

## Supporting information



Supporting Information

## Data Availability

The data that support the findings of this study are available from the corresponding author upon reasonable request.

## References

[1] M. Cinquino , A. Fieramosca , R. Mastria , L. Polimeno , A. Moliterni , V. Olieric , N. Matsugaki , R. Panico , M. De Giorgi , G. Gigli , C. Giannini , A. Rizzo , D. Sanvitto , L. De Marco , Adv. Mater. 2021, 33, 2102326. 34623706 10.1002/adma.202102326 PMC11469044

[2] C. Bao , Z. Yuan , W. Niu , J. Yang , Z. Wang , T. Yu , J. Wang , F. Gao , Nat. Electron. 2024, 7, 375.

[3] Z. Dang , F. Guo , Z. Wang , W. Jie , K. Jin , Y. Chai , J. Hao , ACS Nano 2024, 18, 27727. 39324409 10.1021/acsnano.4c10231

[4] Z. Liu , Y. Fang , Z. Cai , Y. Liu , Z. Dong , R. Zheng , Z. Shen , R. Wu , W. Qu , J. Fu , C. Ru , Y. Wu , J. Gu , Y. Liu , Q. Liu , C. Zhao , Z. Wen , Nano Energy 2024, 132, 110347.

[5] Y. Cao , Y. Fang , L. Yin , Y. Fang , G. Zhu , L. Li , Z. Chen , J. Cao , Y. Liu , C. Zhao , G. Lu , Nano Energy 2025, 139, 110901.

[6] L. Zhang , Y. Chen , S. Mao , Z. Li , C. Jiang , C. Luo , H. Lin , J. Travas‐Sejdic , H. Peng , Chem. Eng. J. 2025, 506, 160106.

[7] J. C. Blancon , H. Tsai , W. Nie , C. C. Stoumpos , L. Pedesseau , C. Katan , M. Kepenekian , C. M. Soe , K. Appavoo , M. Y. Sfeir , S. Tretiak , P. M. Ajayan , M. G. Kanatzidis , J. Even , J. J. Crochet , A. D. Mohite , Science 2017, 355, 1288. 28280250 10.1126/science.aal4211

[8] E. Shi , S. Deng , B. Yuan , Y. Gao , Akriti, L. Yuan , C. S. Davis , D. Zemlyanov , Y. Yu , L. Huang , L. Dou , ACS Nano 2019, 13, 1635. 30812095 10.1021/acsnano.8b07631

[9] C. Zhao , W. Tian , Q. Sun , Z. Yin , J. Leng , S. Wang , J. Liu , K. Wu , S. Jin , J. Am. Chem. Soc. 2020, 142, 15091. 32786774 10.1021/jacs.0c06572

[10] M. Seitz , M. Meléndez , N. Alcázar‐Cano , D. N. Congreve , R. Delgado‐Buscalioni , F. Prins , Adv. Opt. Mater. 2021, 9, 2001875.

[11] S. He , T. Jin , A. Ni , T. Lian , J. Phys. Chem. Lett. 2023, 14, 2241. 36820889 10.1021/acs.jpclett.2c03815 PMC10009813

[12] K. Kobbekaduwa , S. Shrestha , P. Adhikari , E. Liu , L. Coleman , J. Zhang , Y. Shi , Y. Zhou , Y. Bekenstein , F. Yan , A. M. Rao , H. Tsai , M. C. Beard , W. Nie , J. Gao , Nat. Commun. 2021, 12, 1636. 33712623 10.1038/s41467-021-21946-2 PMC7954808

[13] R. H. Bube , Photoelectronic Properties of Semiconductors, Cambridge University Press, Cambrige 1992.

[14] H. Jin , E. Debroye , M. Keshavarz , I. G. Scheblykin , M. B. Roeffaers , J. Hofkens , J. A. Steele , Mater. Horiz. 2020, 7, 397.

[15] A. Musiienko , J. Pipek , P. Praus , M. Brynza , E. Belas , B. Dryzhakov , M. H. Du , M. Ahmadi , R. Grill , Sci. Adv. 2020, 6, 37. 10.1126/sciadv.abb6393 PMC7486106 32917707

[16] F. Winterer , L. S. Walter , J. Lenz , S. Seebauer , Y. Tong , L. Polavarapu , R. T. Weitz , Adv. Electron. Mater. 2021, 7, 6.

[17] B. Wenger , P. K. Nayak , X. Wen , S. V. Kesava , N. K. Noel , H. J. Snaith , Nat. Commun. 2017, 8, 590. 28928482 10.1038/s41467-017-00567-8 PMC5605602

[18] Y. B. Lu , X. Kong , X. Chen , D. G. Cooke , H. Guo , Sci. Rep. 2017, 7, 41860. 28150743 10.1038/srep41860 PMC5288793

[19] I. Amit , T. J. Octon , N. J. Townsend , F. Reale , C. D. Wright , C. Mattevi , M. F. Craciun , S. Russo , Adv. Mater. 2017, 29, 1605598. 10.1002/adma.201605598 28295639

[20] N. J. Townsend , I. Amit , V. Panchal , O. Kazakova , M. F. Craciun , S. Russo , Phys. Rev. B 2019, 100, 165310.

[21] C. R. McNeill , I. Hwang , N. C. Greenham , J. Appl. Phys. 2009, 106, 2.

[22] R. Mastria , K. J. Riisnaes , A. Bacon , I. Leontis , H. T. Lam , M. A. S. Alshehri , D. Colridge , T. H. E. Chan , A. De Sanctis , L. De Marco , L. Polimeno , A. Coriolano , A. Moliterni , V. Olieric , C. Giannini , S. Hepplestone , M. F. Craciun , S. Russo , Adv. Funct. Mater. 2024, 2401903, 1.

[23] K. J. Riisnaes , M. Alshehri , I. Leontis , R. Mastria , H. T. Lam , L. De Marco , A. Coriolano , M. F. Craciun , S. Russo , ACS Appl. Mater. Interfaces 2024, 16, 31399. 38836799 10.1021/acsami.4c02966 PMC11195008

[24] L. Polimeno , G. Lerario , M. De Giorgi , L. De Marco , L. Dominici , F. Todisco , A. Coriolano , V. Ardizzone , M. Pugliese , C. T. Prontera , V. Maiorano , A. Moliterni , C. Giannini , V. Olieric , G. Gigli , D. Ballarini , Q. Xiong , A. Fieramosca , D. D. Solnyshkov , G. Malpuech , D. Sanvitto , Nat. Nanotechnol. 2021, 16, 1349. 34675412 10.1038/s41565-021-00977-2

[25] K. J. Riisnaes , L. De Marco , L. Polimeno , M. Craciun , S. Russo , in Frontiers in Optics/Laser Science, (Eds: B. Lee , C. Mazzali , K. Corwin , R. Jason Jones ), OSA Technical Digest, Optica Publishing Group 2020, paper FTu6B.4.

[26] R. Mastria , K. J. Riisnaes , A. Bacon , I. Leontis , H. T. Lam , M. A. S. Alshehri , D. Colridge , T. H. E. Chan , A. De Sanctis , L. De Marco , L. Polimeno , A. Coriolano , A. Moliterni , V. Olieric , C. Giannini , S. Hepplestone , M. F. Craciun , S. Russo , Adv. Funct. Mater. 2024, 34, 2401903.

[27] B. Saparov , D. B. Mitzi , Chem. Rev. 2016, 116, 4558. 27040120 10.1021/acs.chemrev.5b00715

[28] D. B. Straus , C. R. Kagan , J. Phys. Chem. Lett. 2018, 9, 1434. 29481089 10.1021/acs.jpclett.8b00201

[29] M. Cinquino , A. Fieramosca , R. Mastria , L. Polimeno , A. Moliterni , V. Olieric , N. Matsugaki , R. Panico , M. D. Giorgi , G. Gigli , C. Giannini , A. Rizzo , D. Sanvitto , L. D. Marco , Adv. Mater. 2021, 33, 2102326. 34623706 10.1002/adma.202102326 PMC11469044

[30] X. Gao , X. Zhang , W. Yin , H. Wang , Y. Hu , Q. Zhang , Z. Shi , V. L. Colvin , W. W. Yu , Y. Zhang , Adv. Sci. 2019, 6, 1900941. 10.1002/advs.201900941 PMC6864510 31763136

[31] F. Withers , T. Bointon , D. Hudson , M. Craciun , S. Russo , Sci. Rep. 2014, 4, 4967.

[32] M.‐K. Li , T.‐P. Chen , Y.‐F. Lin , C. M. Raghavan , W.‐L. Chen , S.‐H. Yang , R. Sankar , C.‐W. Luo , Y.‐M. Chang , C.‐W. Chen , Small 2018, 14, 1803763. 10.1002/smll.201803763 30430728

[33] K. Leng , L. Wang , Y. Shao , I. Abdelwahab , G. Grinblat , I. Verzhbitskiy , R. Li , Y. Cai , X. Chi , W. Fu , P. Song , A. Rusydi , G. Eda , S. A. Maier , K. P. Loh , Nat. Commun. 2020, 11, 2. 33127900 10.1038/s41467-020-19331-6 PMC7599242

[34] M. K. Li , T. P. Chen , Y. F. Lin , C. M. Raghavan , W. L. Chen , S. H. Yang , R. Sankar , C. W. Luo , Y. M. Chang , C. W. Chen , Small 2018, 14, 52. 10.1002/smll.201803763 30430728

[35] F. Liu , L. Wang , J. Wang , F. Wang , Y. Chen , S. Zhang , H. Sun , J. Liu , G. Wang , Y. Hu , C. Jiang , Adv. Funct. Mater. 2021, 31, 1.

[36] G. Armaroli , L. Maserati , A. Ciavatti , P. Vecchi , A. Piccioni , M. Foschi , V. Van der Meer , C. Cortese , M. Feldman , V. Foderá , T. Lemercier , J. Zaccaro , J. M. Guillén , E. Gros‐Daillon , B. Fraboni , D. Cavalcoli , ACS Energy Lett. 2023, 8, 4371. 37854053 10.1021/acsenergylett.3c01429 PMC10580305

[37] S. Pandey , C. Biswas , T. Ghosh , J. J. Bae , P. Rai , G. H. Kim , K. J. Thomas , Y. H. Lee , P. Nikolaev , S. Arepalli , Nanoscale 2014, 6, 3410. 24531922 10.1039/c3nr05675a

[38] B. I. Shklovskii , A. L. Efros , Electronic Properties of Doped Semiconductors, Springer Series in Solid State Sciences, vol 45, Springer, Berlin 1984.

[39] T. Khodkov , I. Khrapach , M. F. Craciun , S. Russo , Nano Lett. 2015, 15, 4429. 26079989 10.1021/acs.nanolett.5b00772

[40] F. Withers , S. Russo , M. Dubois , M. F. Craciun , Nanoscale Res. Lett. 2011, 6, 1. 10.1186/1556-276X-6-526 PMC3212065 21910905

[41] H. Qiu , T. Xu , Z. Wang , W. Ren , H. Nan , Z. Ni , Q. Chen , S. Yuan , F. Miao , F. Song , G. Long , Y. Shi , L. Sun , J. Wang , X. Wang , Nat. Commun. 2013, 4, 3. 10.1038/ncomms3642 24149969

[42] A. L. Efros , B. I. Shklovskii , Electron‐Electron Interactions in Disordered Systems, vol. 409, North‐Holland, Amsterdam 1985.

[43] J. Lee , J. B. Ferguson , A. M. Hubbard , Y. Ren , D. Nepal , T. C. Back , N. R. Glavin , A. K. Roy , Mater. Today Commun. 2024, 39, 1.

[44] E. Piatti , A. Arbab , F. Galanti , T. Carey , L. Anzi , D. Spurling , A. Roy , A. Zhussupbekova , K. A. Patel , J. M. Kim , D. Daghero , R. Sordan , V. Nicolosi , R. S. Gonnelli , F. Torrisi , Nat. Electron. 2021, 4, 893.

[45] J. Lee , J. B. Ferguson , A. M. Hubbard , Y. Ren , D. Nepal , T. C. Back , N. R. Glavin , A. K. Roy , Mater. Today Commun. 2024, 39, 108859.

[46] S. M. Sze , K. K. Ng , Physics of Semiconductor Devices: Third Edition, John wiley & Sons, New Jersey 2006.

[47] A. M. Goodman , A. Rose , J. Appl. Phys. 1971, 42, 2823.

[48] R. Xiao , Y. Hou , Y. Fu , X. Peng , Q. Wang , E. Gonzalez , S. Jin , D. Yu , Nano Lett. 2016, 16, 7710. 27960528 10.1021/acs.nanolett.6b03782

[49] V. D. Mihailetchi , J. Wildeman , P. W. Blom , Phys. Rev. Lett. 2005, 94, 1. 10.1103/PhysRevLett.94.126602 15903944

[50] S. Shrestha , X. Li , H. Tsai , C. H. Hou , H. H. Huang , D. Ghosh , J. J. Shyue , L. Wang , S. Tretiak , X. Ma , W. Nie , Chem 2022, 8, 1107.

[51] R. Graham , D. Yu , Mod. Phys. Lett. B 2013, 27, 1.

[52] J. D. Mehew , S. Unal , E. Torres Alonso , G. F. Jones , S. Fadhil Ramadhan , M. F. Craciun , S. Russo , Adv. Mater. 2017, 29, 23. 10.1002/adma.201700222 28418620

[53] F. Guo , B. Yang , Y. Yuan , Z. Xiao , Q. Dong , Y. Bi , J. Huang , Nat. Nanotechnol. 2012, 7, 798. 23142945 10.1038/nnano.2012.187

[54] R. Dong , Y. Fang , J. Chae , J. Dai , Z. Xiao , Q. Dong , Y. Yuan , A. Centrone , X. C. Zeng , J. Huang , Adv. Mater. 2015, 27, 1912. 25605226 10.1002/adma.201405116

[55] Y. Fan , Y. Zhou , X. Wang , H. Tan , Y. Rong , J. H. Warner , Adv. Opt. Mater. 2016, 4, 1573.

[56] R. J. Chang , H. Tan , X. Wang , B. Porter , T. Chen , Y. Sheng , Y. Zhou , H. Huang , H. Bhaskaran , J. H. Warner , ACS Appl. Mater. Interfaces 2018, 10, 13002. 29630341 10.1021/acsami.8b01038

[57] S. F. Soares , Japanese J. Appl. Phys. 1992, 31, 210.

[58] A. De Sanctis , G. F. Jones , N. J. Townsend , M. F. Craciun , S. Russo , Rev. Sci. Instrum. 2017, 88, 5. 10.1063/1.4982358 28571447

[59] V. Podzorov , V. Bruevich , Nat. Electron. 2024, 7, 266.

