# Supplementary material for: Decoding Trap States in Working 2D Perovskite Multi‐Functional Devices
Source: Adv Sci (Weinh). 2026 Jan 12;13(12):e18675. doi: 10.1002/advs.202518675 (PMC12948258; doi:10.1002/advs.202518675)
Supplement: Supplementary file 1 — Supporting Information [file ADVS-13-e18675-s001.pdf]

Supporting Information: Decoding Trap States in Working 2D Perovskite multi-functional devices

Ioannis Leontis,† Karl Jonas Riisnaes,† Hoi Tung Lam,† Rosanna Mastria,‡ Luisa De Marco,‡ Annalisa Coriolano,‡ Steven Hepplestone,† Monica Felicia Craciun,† and Saverio Russo*,†

†*Centre for Graphene Science, College of Engineering, Mathematics and Physical Sciences, University of Exeter, Exeter EX4 4QL, United Kingdom*

‡*CNR NANOTEC, Institute of Nanotechnology, via Monteroni, 73100, Lecce, Italy*

E-mail: s.russo@exeter.ac.uk

S0. Electrodes fabrication, 2D F-PEAI lamination, photoluminescence and electronic structure

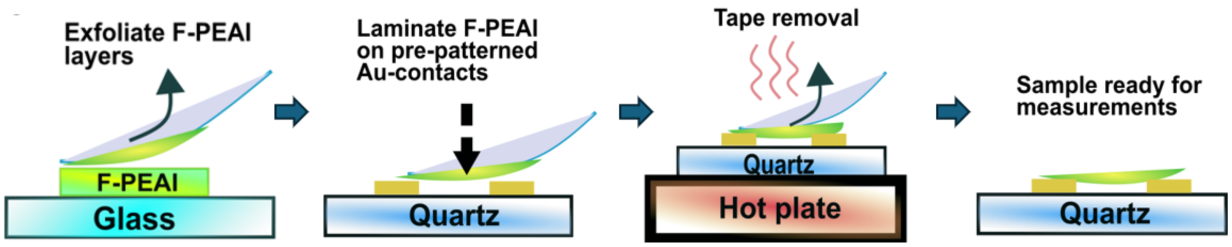

Fig. S0: Diagram showing the process exfoliating F-PEAI flakes onto a pre-patterned quartz substrate.

Key fabrication steps. Standard electron beam lithography (e-beam lithography) is used to define the contact patterns for 2D F-PEAI photodetectors. A positive-tone polymethyl methacrylate (PMMA) resist is spin-coated onto the substrate and baked to form a uniform resist layer. The desired contact patterns are written into the PMMA layer using a focused electron beam. After exposure and development of the resist, the exposed areas are metallized using electron beam deposition in a system with low background pressure ($< 2 \times 10^{-8}$ Torr) and a cooled substrate stage (20°C). Following the lift-off process, metalized electrodes and bonding pads are left on the substrate. Figure S0 shows the process of transferring F-PEAI flakes onto a pre-patterend quartz substrate. The F-PEAI flakes are grown on a glass substrate, and exfoliated using using thermal release tape (Graphene Supermarket, SKU: GTT-5P). Hence, the crystals are laminated onto pre-patterned Au contacts and transferred by heating at 95^oC on a hotplate.

Interdigitated electrodes. In the devices presented in this work, we employ an interdigitated electrode (IDE) geometry, which is particularly well-suited for 2D materials such as 2D F-PEAI (see Fig. S1a). The choice of this geometry is motivated by several considerations that collectively enhance the performance and reliability of optoelectronic measurements. First, IDEs significantly increase the electrode surface area in contact with the active semiconducting film, without enlarging the device footprint. This expanded con-

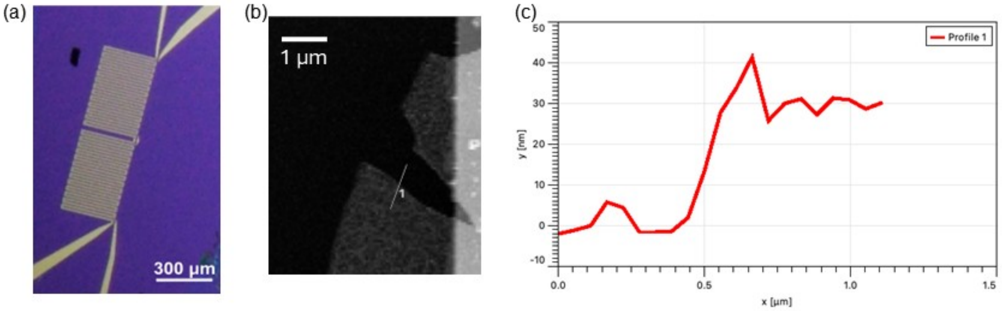

Fig. S1: (a) Micrograph image of the interdigitated electrode geometry used in this work. (b-c) Atomic Force Microscopy image of the 2D F-PEAI and corresponding plot for the line cut marked as (1), respectively.

tact interface is critical for minimizing contact resistance, which scales inversely with the contact area,[1–4] The short spacing between adjacent fingers ensures that photo-generated carriers traverse minimal distances before collection, which is especially important for materials where the carrier diffusion length is comparable to or smaller than the electrode spacing. The probability of recombination during transport increases with the square of the transit time, and thus minimizing the transit path length significantly improves collection efficiency and it boosts the time response of photodetectors.[2,5] Third, the IDE design allows for a high degree of reproducibility and sensitivity in photodetection. The geometry provides multiple parallel conduction paths, improving the signal-to-noise ratio (SNR) by averaging out local material non-uniformities or defects. This redundancy is beneficial for both steady-state and transient measurements. Finally, from a practical standpoint, the IDE layout provides an ideal balance between scalability, device integration, and compatibility with planar fabrication processes. It is especially well-suited for 2D materials, where traditional vertical device architectures may introduce unwanted strain or damage to the active layer. Fig. S1 (b-c) show the Atomic Force Microscopy image for 2D F-PEAI and the corresponding line cut.

Temperature dependence of photoluminescence. Optical properties of 2D perovsites are strongly related to their crystal symmetry and therefore they are directly affected by temperature induced phase transitions.[6–8] Changes in crystal symmetry affect the band structure, and can result in the lifting of degeneracies, modification of orbital overlap, or

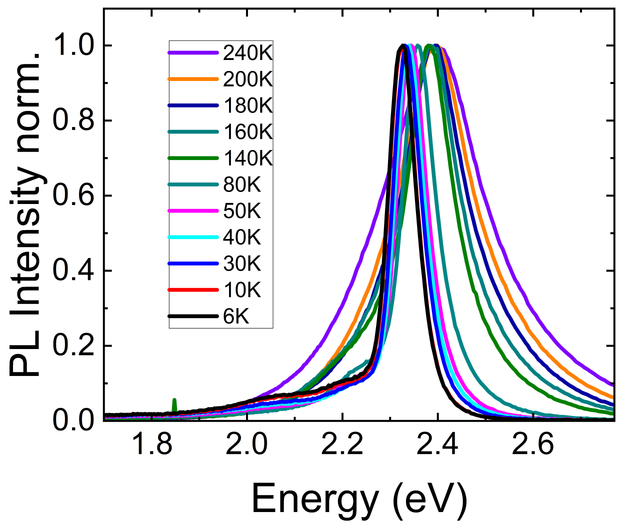

Fig. S2: Plot of the temperature dependence of the photoluminescence measured in 2D F-PEAI using laser light $\lambda = 375nm$, see methods.

emergence of new electronic states. These changes are often manifested in discrete jumps in emission energy and the appearance of multiple emissive features in photoluminescence with temperature.[9,10] In contrast, our measurements reveal a gradual redshift of the PL peak with decreasing temperature, which is consistent with bandgap widening due to thermal lattice contraction and reduced electron–phonon coupling—both expected effects in the absence of any structural phase change. Fig. S2 shows the measured PL spectra for 2D F-PEAI over a wide temperature range. Our measurements reveal a gradual redshift of the PL peak with decreasing temperature, likely driven by dielectric screening or exciton localization effects as reported by other authors[11],[12] and a band gap narrowing due to thermal contraction, rather than a crystallographic transition. Similarly, the widening of the emission band upon increasing temperature is due to enhanced electron–phonon coupling expected in the absence of any structural phase change. This behaviour is well-documented in many semiconductors and reflects the expected influence of thermal expansion (and contraction) on the band structure, rather than a crystallographic transition.

Electronic structure calculations. To assess the role of thermal expansion and strain on the structures, we have carried out a systematic study of the variation of the electronic

gap using the GGA functional. This functional is known to underestimate the bandgap, but to provide systematic errors as a function of strain. Thus we present our variation of the bandgap as a percentage, rather than as absolute values. The GGA bulk experimental band gap is 2.17 eV. Our results show that decreasing strains of up to -5% can result in changes in the band gap of double the magnitude, resulting in a substantial shift in the band gap. Given that hybrid perovskite structures have a range of thermal expansion coefficients ranging from 3×10^{-5} to 5×10^{-4}[?] (which are equivalent to 0.75% to 12.5% in the temperature range considered) this indicates that these 2D structures undergo thermal expansions of a comparable degree.

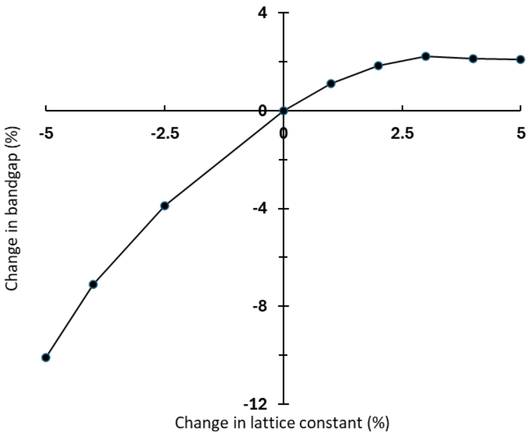

Fig. S3: Plot of the calculated variation in the electronic gap of 2D F-PEAI with changing lattice constant calculated using density functional theory with the PBE GGA functional.

In this work, first-principles techniques based on density functional theory (DFT) were used to determine the structural and electronic properties of FPEAI, see Fig.S3. These calculations were performed using the Vienna ab initio simulation package (VASP) following the steps described in the supplementary information by Mastria et al.[2] The valence electrons modeled with the PAW Pseudopotentials for Pb, I, C, H, F and N were 4, 7, 4, 1, 7, and 5 respectively. The projector augmented wave method was used to describe the interaction between core and valence electrons, and a plane-wave basis set was used with an energy cutoff of 600 eV. All calculations were completed using the Perdew-Burke-Ernzerhof (PBE) generalised gradient approximation functional,[13] and with spin-polarised settings.

Structures were relaxed using GGA-PBE with a fixed unit cell size compared to the experimental bulk. The unit cell sizes were strained uniformly from -5% to +5%. For geometric relaxations, all forces were relaxed to below 0.01 eV/Å per atom, with electronic self-consistency converged to 10^{-7} eV. Atomic positions were allowed to relax.

S1. Transfer characteristic of F-PEAI FET and leakage current of 2D F-PEAI FET

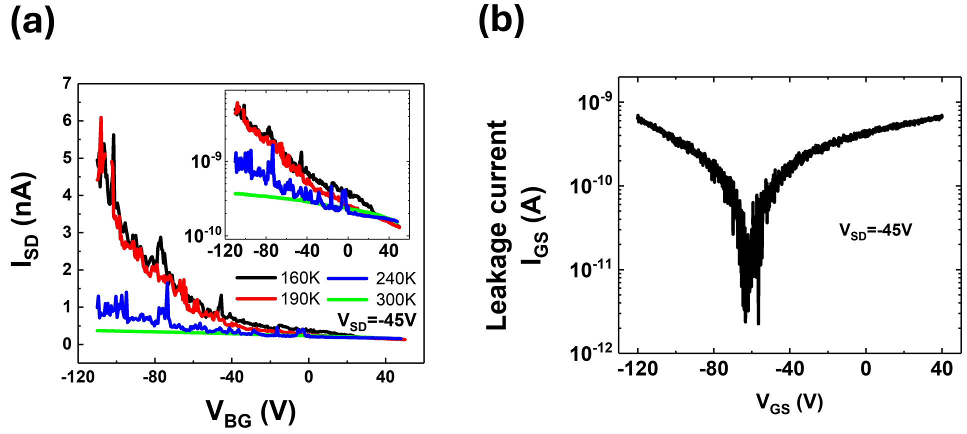

Fig. S4: Temperature dependent transfer characteristic of 2D F-PEAI FET for fixed $V_{SD} = -45V$ for T>100K, (b) Characteristic leakage current (I_{GS} with V_{SD}=45V.

S2. Fowler–Nordheim plot of F-PEAI FET at $V_{BG} = -80V$ and T=4.2K

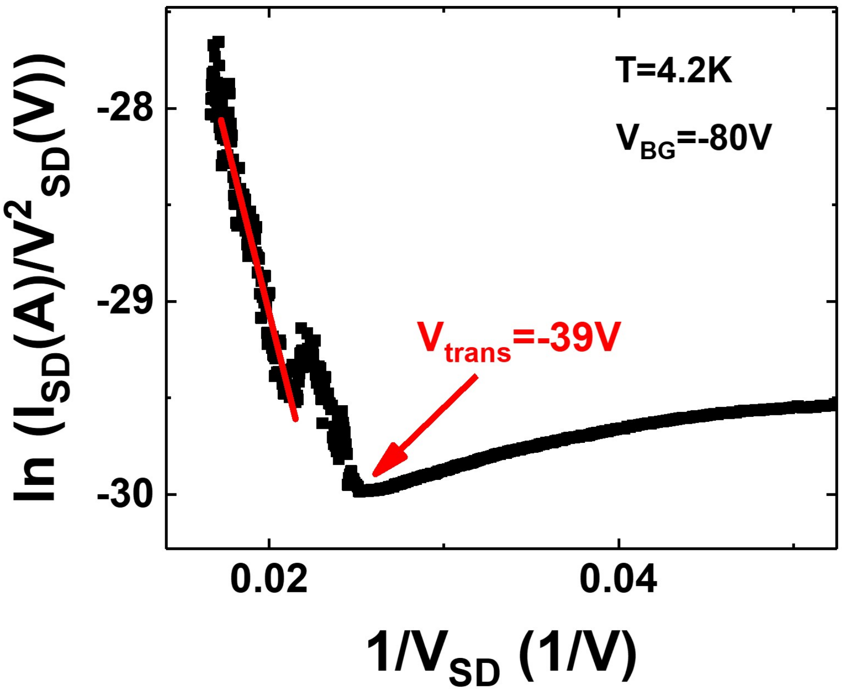

Fig. S5: Fowler–Nordheim plot of F-PEAI FET at $V_{BG} = -80V$ and $T = 4.2\,\mathrm{K}$ where V_{trans} is the bias voltage at which the transition from direct to Fowler–Nordheim tunneling occurs. The red line shows the linear fit of the experimental data at that voltage range

S3. Nernst–Einstein plot of F-PEAI FET at $V_{BG} = -100V$

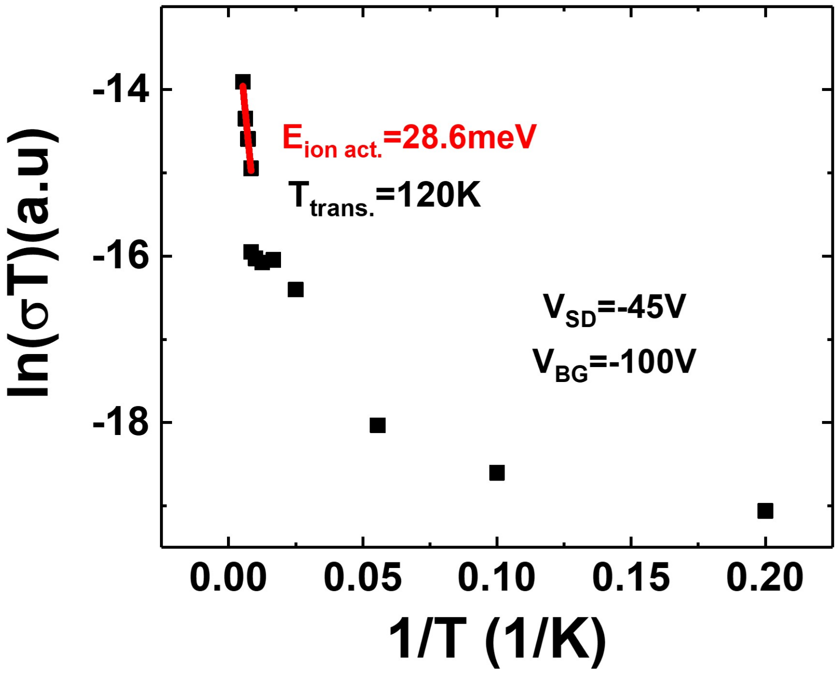

Fig. S6: Nernst–Einstein plot of F-PEAI FET at $V_{BG} = -100V$ where E_α is the activation energy of the ionic movement and T_{trans} is the temperature at which $ln(\sigma T)$ is linear *vs.* $1/T$ indicating the transition temperature from pure electronic to mixed ionic and electronic transport.

The ionic contribution to the electrical conductivity (σ) can be described by the Nernst–Einstein relation, $\sigma(T) = (\sigma_0/T)exp(E_\alpha/k_B T))$, where k_B is the Boltzman constant, and E_α the activation energy. The Nernst–Einstein plot, $ln(\sigma T)$ vs$(1/T)$ shows linear behavior for T>120K indicating a transition temperature for the ionic movement of $\approx 120\,\mathrm{K}$ and an activation energy E_α=28.6meV, respectively (see Figure S6). As the temperature increases $>120\,\mathrm{K}$, the ionic conduction progressivly increases and eventually screens the field effects modulation.

S4. Temperature dependence of variable range hopping in 2D and band conduction.

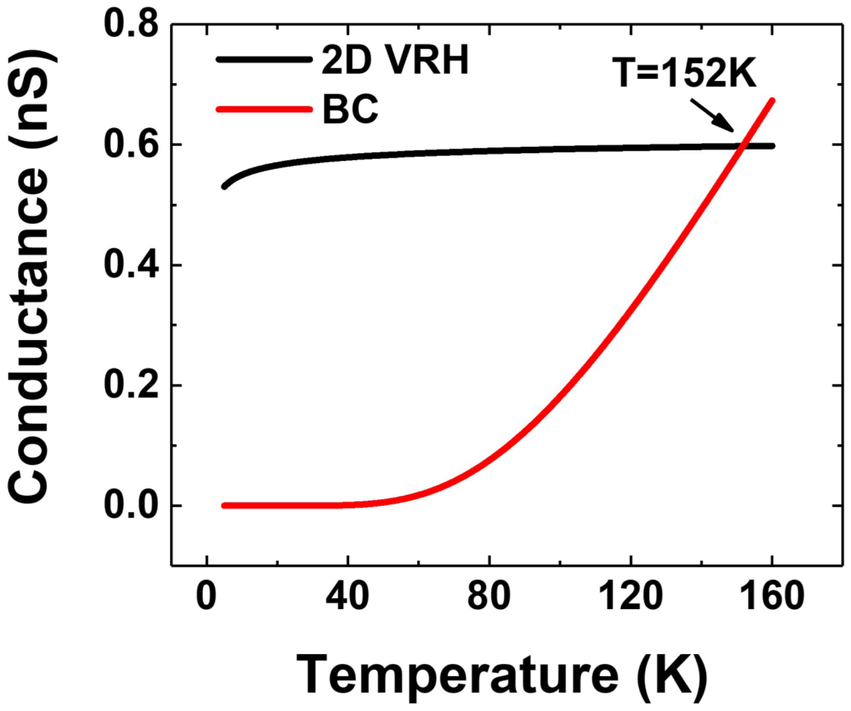

Fig. S7: Temperature depedence of 2D VRH an BC conductance in 2D F-PEAI.

Depending on the level of disorder, the transport of charge carriers at low temperatures can occur via variable range hopping (VRH) through localized states. The temperature dependence of the conductivity in this regime follows an exponential law with the power of the exponent bearing direct information on the dimensionality of the hopping process. More specifically, for a two-dimensional system such as 2D F-PEAI, the conductance VRH conductance is given by $\sigma_{2D-VRH} = \sigma_0 e^{-(E_\alpha/k_B T)^{1/3}}$, with E_α an activation energy. The $T^{-1/3}$ exponent is specific to 2D systems. As the temperature decreases, carrier transport becomes increasingly reliant on tunneling between localized states, influenced by both their spatial distribution and the energy barriers, see Figure S2. This distinct behavior is a hallmark of disordered systems and provides critical insight into the role of defect states in charge

transport. The presence of trap states is also evident from the observation of large hysteresis in the low temperature transfer characteristics for 2D F-PEAI transistors, see Figure S6.

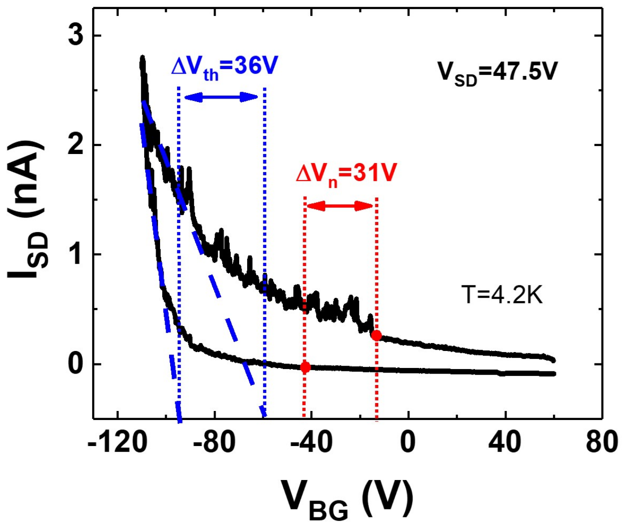

Fig. S8: (a) Hysteresis of 2D F-PEAI FET at T=4.3K for V_{SD}=-47.5V presenting the shift of the threshold voltage (ΔV_{th}) and of the neutrality point (ΔV_n) during the up-down sweaps of the back gate voltages (V_{BG}). Neutrality point, V_n, is actually the V_{BG} when the channel is charge neutral, while V_{th} is actually the V_{BG} when channel in ON, meaning concentration of carriers increases linearly with back gate field effect.

On the other hand, band conduction (BC) typically becomes the dominant charge transport mechanism at sufficiently high temperatures when the thermal energy of carriers overcomes the energy barriers separating localized states. In this regime, charge can occupy states in the energy bands. The BC conductivity typically follows an Arrhenius-like temperature dependence $\sigma_{BC} = \sigma_0' e^{-(E_\alpha'/k_B T)}$. This temperature functional dependence reflects thermally activated transport, where charge carriers are excited into extended states within the conduction or valence band, enabling efficient mobility. In this case, the activation energy E_α provides a direct measure of the energy barrier separating localized states from the conduction band edge, offering valuable insights into the degree of disorder and the energetic landscape of the material. In the temperature range where both charge transport mechanisms are at play, one can describe the conductance with a multi-channel model as $\sigma tot = \sigma_{2D-VRH} + \sigma_{BC}$, with best fit parameters $E_\alpha/k_B = 0.3K$, $E_\alpha'/k_B = 350K$, $\sigma_0 = 6.32 \times 10^{-10}S$ and $\sigma_0' = 6 \times 10^{-9}S$.

Figure S2 shows the temperature dependence of the corresponding conductance of each transport mechanism up to 180K. For T<152K 2D-VRH is the dominant charge transport mechanism, while for T>152K BC becomes preponderant on the overall conductance of the 2D F-PEAI.

S5. Temperature dependent transfer characteristics of additional 2D F-PEAI FET

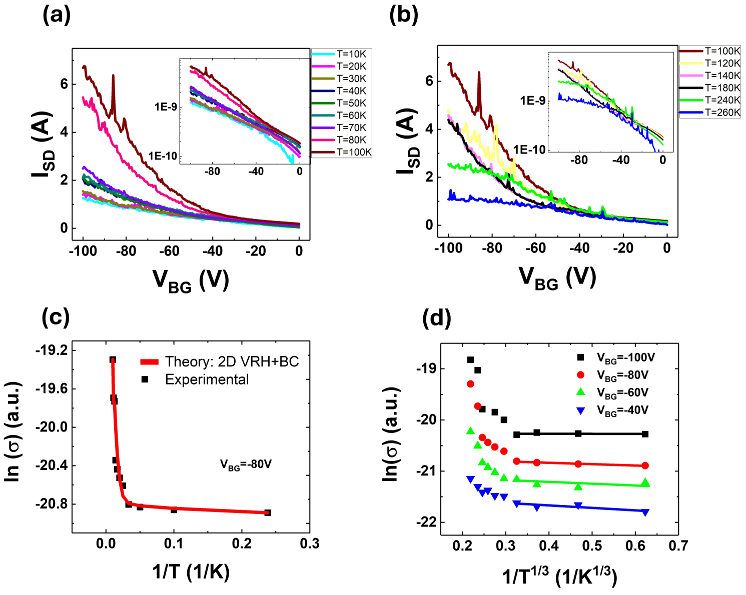

Fig. S9: Temperature dependent transfer characteristic of an extra 2D F-PEAI FET for fixed $V_{SD} = -45V$ for (a) 10K<T<100K, and (b) T>100K. (c) Arrhenius plot of the logarithm of the conductivity as a function of 1/T . The square points are experimental data while the red line is the best fit with the multi-channel conductivity, see main text, and (d) 2D VRH scaling plots for four different values of V_BG

S6. Emission signal for 2D F-PEAI FET at 100K for high V_{SD}

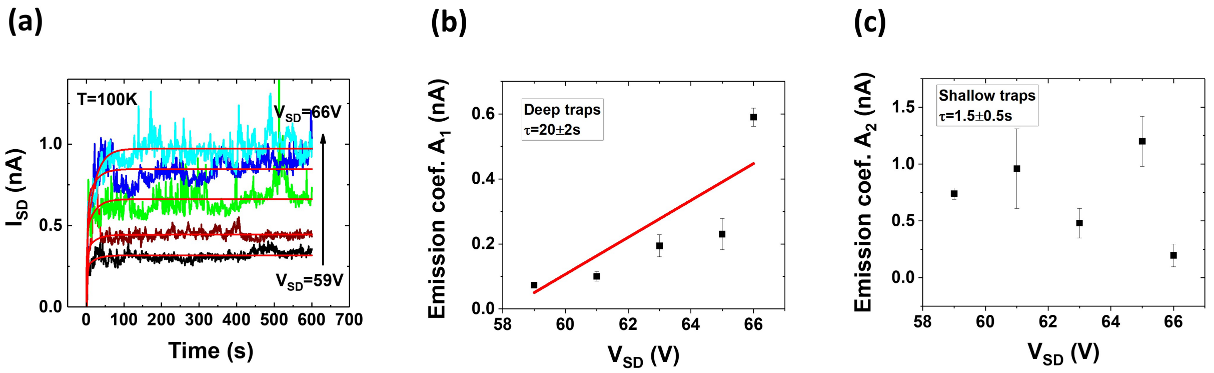

Fig. S10: (a) Time evolution of current during the emission mode of the traps after ON-OFF back gate pulsing (-60 V, 0 V) at 58 V$\leq V_{SD} \leq$ 66 V at 100 K. The red curves are fits of the experimental data (shown in other colours) with double exponential rise equation $I(t) = I_0 + A_1 e^{-t/\tau_1} + A_2 e^{-t/\tau_2}$. (b) and (c) Show plots of the pre-exponential emission coefficients A_1 and A_2 as a function of V_{SD} at 100 K. The red line represents the best linear fit at of A_1.

S7. TVTS, additional TVTS data and fit procedure

Assumptions of TVTS and their validation. Transient threshold voltages spectroscopy (TVTS) monitors the impact of trapping-detrapping through the voltage threshold shifting of the 2D source-drain channel. During the ON-OFF pulsing of a 2D channel V_{th} is shifting from high to low values increasing the source-drain current (I_{sd}) until it reaches a saturation point. The analysis of the time evolution of the drain current during the abrupt ON-OFF controlled by a back gate voltage pulse can give valuable information about the dynamics and the energy of the traps (deep or shallow traps) through a simple fit to exponential rise equation as long as the following conditions are met. (1) Dominance of Threshold Voltage Change. The transient is primarily governed by the V_{th} shifts due to trapped charges and not due to transient emission current. The two different cases are easily distinguishable as the emission current

from charge traps in the case when the Fermi level is less than the Vth (depleted channel) is described by a decay exponential, whereas transient current of a depleted channel with dominant time-depedent changes in the threshold voltage of the 2D channel is described by the rise exponential equation (see equations and discussion in the main article). (2) Uniform Distribution of Trapped Charges across the entire conductive channel. This assumption is supported by the observed shift in the charge neutrality point (see Fig. S6) and the linear dependence of the pre-exponential factors on drain bias for deep traps as well as prior studies of highly spatially resolved photocurrent spectroscopy.[2] (3) No Net Injection of Charges Through Contacts. This implies no net injection of charges through the contacts during the measurement. This assumption is verified by the output characteristic of the samples. Plots in Figure 2 show no drain current when Vbg=0V indicating no injection of carriers through contacts. Moreover, the Fowler-Nordheim plots also show that when Vbg=0V there is no field emission for Vsd<100V. Thus, during the OFF pulse (Vbg=0V) there is no injection of carriers in the 2D channel. (4) Shockley-Reed-Hall kinetics is directly evidenced by the time-dependent density of occupied traps with exponential decay form of their dynamics. This is the case when the transient drain current follows the rise exponential behaviour during the OFF back gate pulse and, also, its pre-exponential factor presents linear voltage dependence (see Eq. 1 in the main manuscript). (5) Channel thickness much smaller than the Debye length. This ensures that the threshold voltage is strongly modulated by space charge regions at the semiconductor-dielectric and semiconductor-ambient interfaces. In other words, trapped charges can be treated as inducing a uniform shift in the Fermi level across the channel, modifying V_{th} globally. This is why the threshold voltage transient model depends only on the total trapped charge density and not on a spatially resolved depletion width. This last assumption is what makes TVTS uniquely suited to characterise trap states in 2D materials, see Table S1.

Detailed Derivation of the time evolution of the density of the occupied traps during the OFF-mode of the back gate pulse. The general case including thermal

assisted emission and re-capture of carriers during the detrapping process. In this paragraph the time evolution of the occupied hole traps close to the valance band following Shockley-Read-Hall statistics is presented. The rate of capturing holes from the valance band (R_{pc}) is proportional to the density of holes in the valance band (p) and the density of unoccupied traps ($N_T - p_T$) according to the relation

$$R_{pc} \equiv \frac{\partial p}{\partial t}\Big|_{capture} = c_p p(N_T - p_T)$$

where N_T is the total density of trapping states and p_T is the density of occupied states, c_p is the capture coefficient for holes, and it equals the thermal velocity, v_{th} , multiplied by the capture cross section, σ_p .

The emission of holes from the traps is described using the same considerations without taking into account the unoccupied states in the valance band, since it is assumed that for a non-degenerate semiconductor the emission rate is not limited by it, with

$$R_{pe} \equiv \frac{\partial p}{\partial t}\Big|_{emission} = -e_p p_T$$

where e_p is the emission rate of holes from traps to the valance band. It is therefore clear, that the total change in trap occupation is given by:

$$R_p = \frac{dp}{dt} = \frac{\partial p}{\partial t}\Big|_{capture} - \frac{\partial p}{\partial t}\Big|_{emission} = c_p p(N_T - p_T) - e_p p_T = -p_T(c_p p + e_p) + c_p p N_T$$

the solution of which is:

$$p_T(t) = p_T(\infty) + [p_T(0) - p_T(\infty)]e^{-(t/\tau)}$$

where $p_T(\infty) = [c_p p/(e_p + c_p p)]$ is the density of the occupied hole traps during the steady state of the low injection level, $p_T(0)$ is the density of the occupied traps before the pulse

and it is equal to N_T if traps are completely filled (saturated) before the pulse, and τ is the decay time of the traps equal to $\tau = 1/(c_p p + e_p)$.

Here two different cases can be distinguished. a) The low injection mode (OFF-mode of the back gate pulse) occurs at $V_{BG} = 0V$ corresponding to a depleted channel at which p is zero. In this case, p=0 and the retrapping effect is significantly reduced. Therefore, $p_T(\infty) = 0$ and $p_T(0) = N_T$ that can simplify $p_T(t)$ to $p_T(t) \sim N_T e^{-(t/\tau)}$. This corresponds to the case in which pure thermal assisted emission of carriers is dominant and it is equivalent to the decay of the traps after a light pulse. b) When $V_{BG} \neq 0V \rightarrow p \neq 0$ retrapping re-capture process of carriers at the traps is active during the detrapping and $p_T(t)$ cannot be simplified. Thus, if the the OFF-mode of the back gate pulse is not zero re-capture of carriers to the traps is enhanced and strong re-trapping effect is dominant during the decay of the traps. Strong retrapping effect induces fluctuations at the log-log plot of the density of the occupied traps *vs* time at the case of discrete trappping centers[14] that is also evident at the log-log plot of the transient I_{SD} of a 2D F-PEAI FET with V_{BG} pulse from -90V to -20V. However, this fluctuations disappear during the pure emission of carriers when V_{BG} pulse is from -90V to 0V as it is evident at Figure S10 .

Additional data. Additional temperature dependent field effect measurements and V_{GS} pulsed measurements were repeated on various samples. here we show a complete set for another additional sample (2D F-PEAI FET). Figures S9.(a) and (b) show plots of the measured temperature dependence of the transfer curve. Similarly to the data for the other device discussed in the main text, we observe p-type conductivity, and the drain current decreases for temperatures >100K due to ions migration resulting in the screening of the field effect.

Figure S9.(c) and (d) presents the emission signal of the new sample after using the same dynamic window of the back gate pulsing $V_{GS} = \breve{}60V$ ("ON") and $V_{GS} = 0V$ ("OFF")) at 4.2K and 100K, respectively. Similarly to the data discussed in the main text, the emission signal is fitted very consistently with the single and double exponential rise equation at

4.2K and 100K, respectively. The pre-exponential factor A_1 has a linear dependence on the source-drain bias at 4.2K and 100K (Figures S2.(e-f)) The best fit to a double exponential at 100K $I(t) = I_0 + A_1 e^{-t/\tau_1} + A_2 e^{-t/\tau_2}$ and the non linear V_{SD} dependence of the emission coefficient A_2 indicate the presence of shallow trapping effects . Similarly to the main text, the fittings of the transient emission current with the single and double exponential rise equation was conducted without pre-assumptions on the pre-exponential coefficients $A_1$and A_2.

Fit procedure. The actual fitting method that was used, to identify the correct number of the exponents during the rise exponential fitting, follows three basic steps that are also presented in Figure S11.a-c and S11.d-f for 100K and 4.2K, respectively. At first, a fit with a single rise exponential function ($I(t) = I_0 - A_1 e^{-t/\tau_1}$) is used to fit the transient I_{SD} during the OFF pulse (see Figure S7.a at 100K and S7.d at 4.2K). Then, the fitting procedure is repeated iteratively adding an additional exponential term every time. Figures S7.b and S7.e present the fit with a double rise exponential function ($I(t) = I_0 - A_1 e^{-t/\tau_1} - A_2 e^{-t/\tau_2}$) while Figures S7.c and S7.f show the fit with a tripe rise exponential function ($I(t) = I_0 - A_1 e^{-t/\tau_1} - A_2 e^{-t/\tau_2} - A_3 e^{-t/\tau_3}$) for both temperatures, as well. The best fit is reached by the minimum number of exponentials with the best chi-square and with clearly distinct detrapping times. As is shown, at 4.2K all the fittings present equal number of chi-square, 0.82, however, all the fits indicate the same detrapping time ($\tau_1 = \tau_2 = \tau_3 = 163s$) meaning that they all correspond to the same trap centers as $I(t) = I_0 - A_1 e^{-t/\tau_1} - A_2 e^{-t/\tau_1} - A_3 e^{-t/\tau_1} = I_0 - (A_1 + A_2 + A_3) e^{-t/\tau_1}$. Besides, at 100K, fitting with the double rise exponential equation gives higher chi-square rather than that of single and triple exponential rise equation at this temperature. Additionally, during the fitting with the triple exponential rise equation the detrapping time of the second and third exponential factor is the same ($\tau_2 = \tau_3 = 20s$) confirming further that only two different trapping centers correspond to the time evolution of I_{SD} at this temperature, in direct contrast to only one trapping center at 4.2K. All the used values of the emission coefficients A_1, A_2 and the detrapping time τ_1, τ_2 and their error

range were extracted by the fitted parameters of the best-chosen fitting using the above method. Direct evidence of a double exponential rise time in the high temperature data is garnered by presenting the experimental data with the best fit to a single and double exponential rise equation, see Fig. 11-12. It is apparent that the single exponential failes to accurately describe the data in both the short and long time scale of the trap dynamics. This is due to the presence of two very different trap states (shallow and deep) with very different dynamics, which can only be captured with a double exponential rise equation.

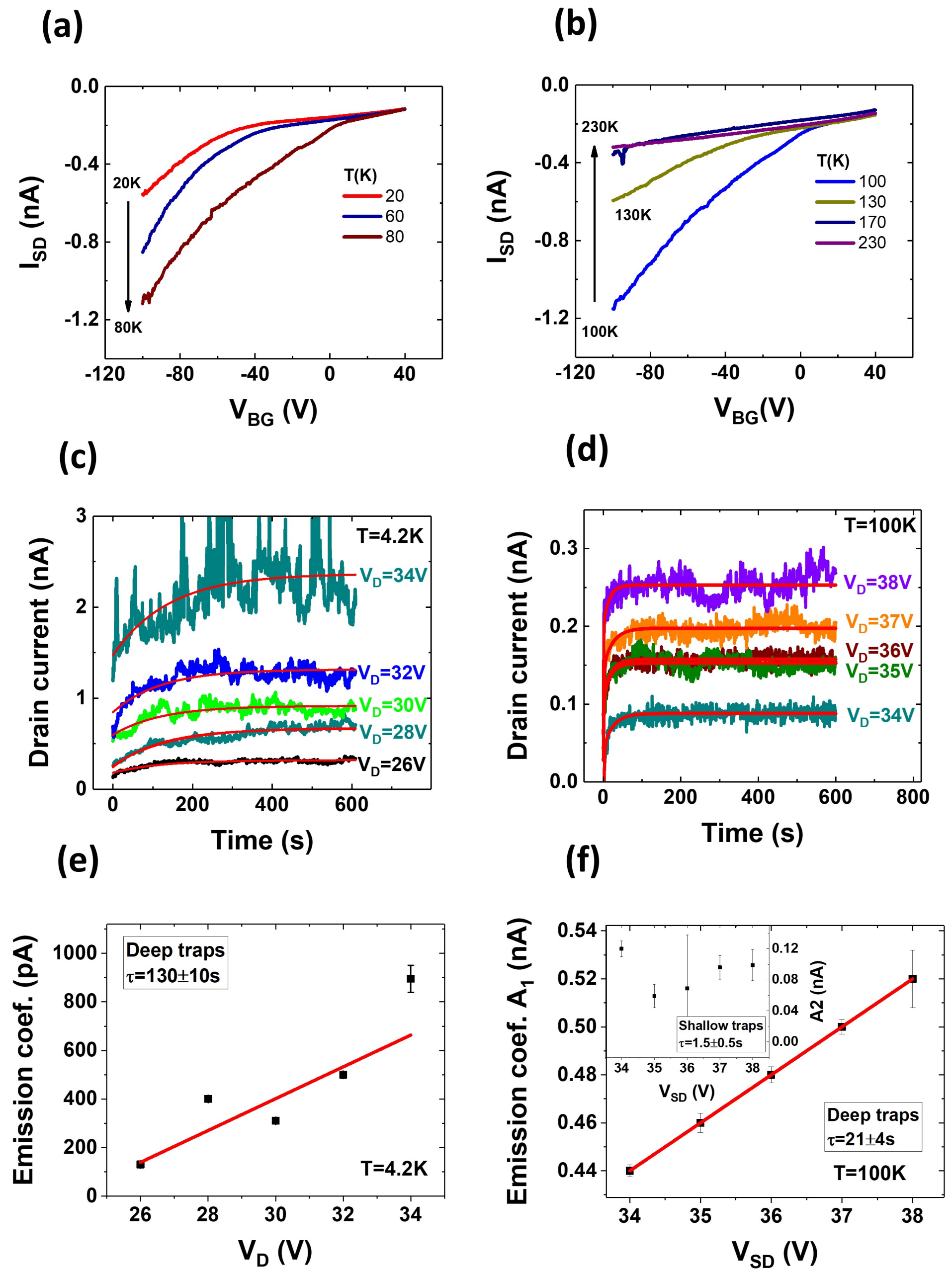

Fig. S11: (a) (b) Transfer characteristics of another 2D F-PEAI FET than those discussed in the main manuscript at various temperature (from $T = 5\,\mathrm{K}$ to $230\,\mathrm{K}$).(c) and (d) show the time evolution of current during the emission mode of the traps after ON-OFF back gate pulsing (-60V, 0V) at various V_{SD} at 4.2 K and 100 K, respectively. The red curves are the fit of the experimental data (shown in other colours) using a single exponential. (e) and (f) are plots of the pre-exponential emission coefficients A_1 and A_2 as a function of V_{SD} at 4.2 K and 100 K, respectively. The red line represents the best linear fit at 4.2K.

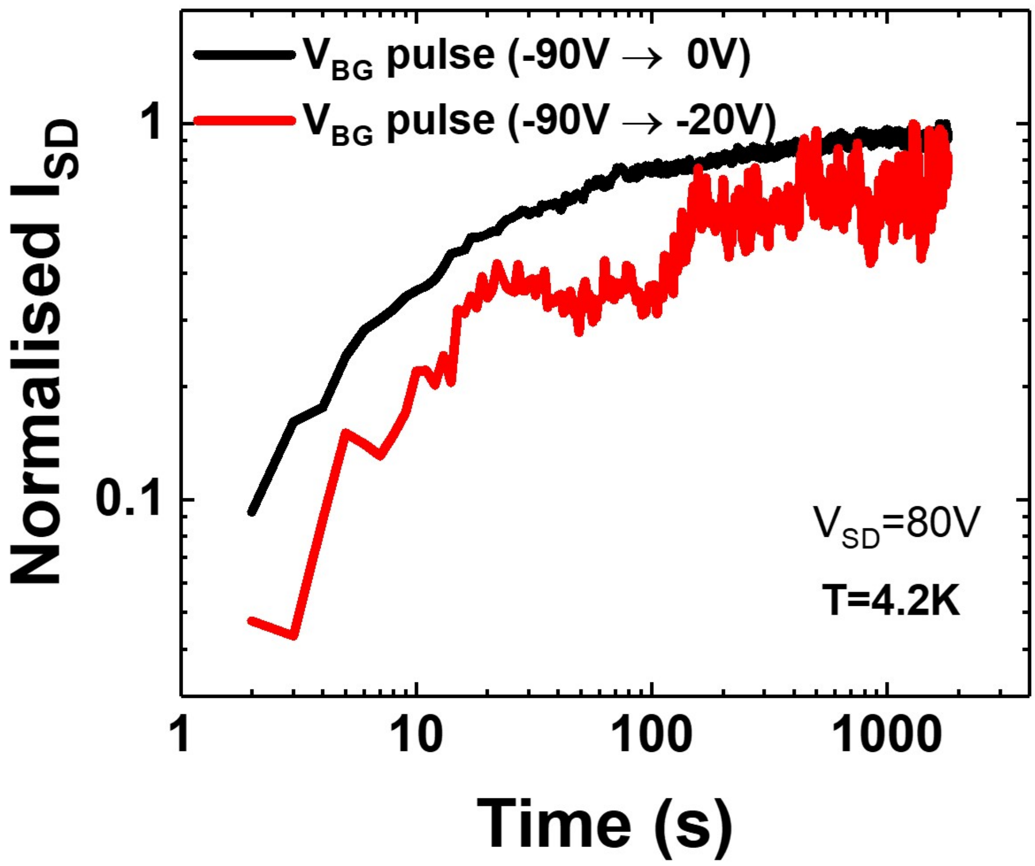

Fig. S12: Log-log plots of the normalised transient I_{SD} vs time during the OFF-back gate pulse of a 2D F-PEAI FET with discrete trapping centers when V_{BG} pulse from -90V to 0V (black line) and from -90V to -20V (red line). During the back gate pulse from -90V to -20V flactuations due to strong retrapping effect is evident that they desappear durring the pure emission decay when V_{BG} pulse is from -90V to 0V

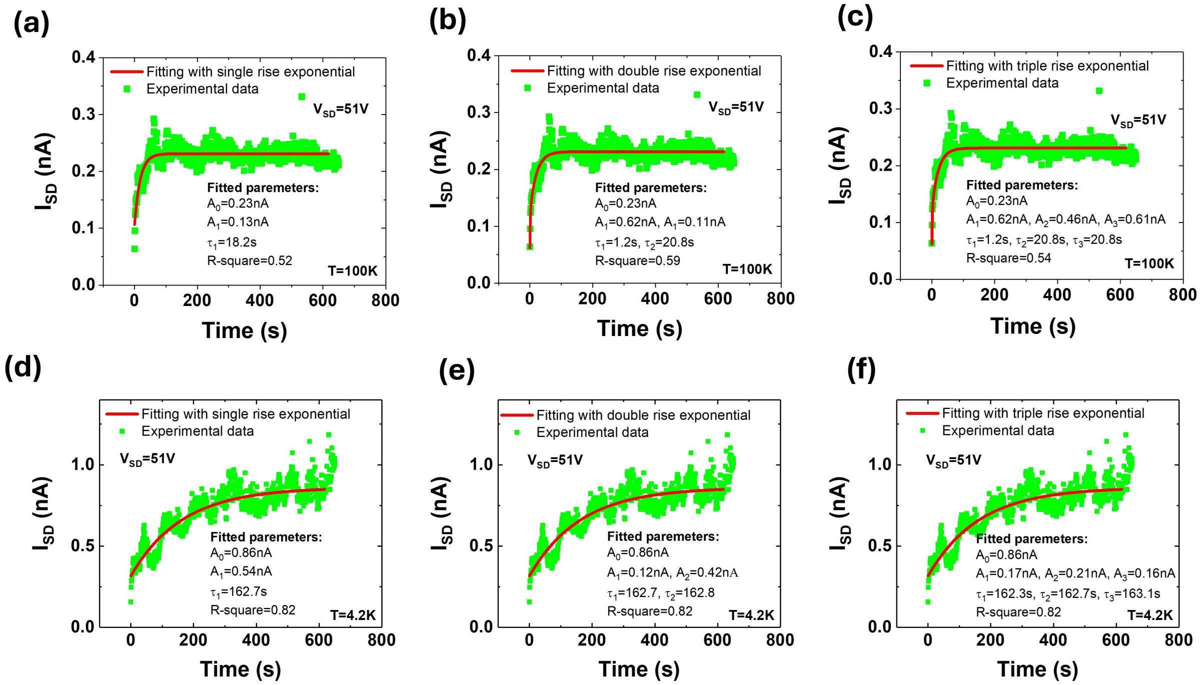

Fig. S13: Fittings of time evolution of the I_{SD} during the emission mode of the traps (OFF back gate pulse) when V_{SD}=51V at 100K a), b), c) and at 4.2K d), e), f). The red curves are fits of the experimental data (shown in green dots) with single exponential rise equation $I(t) = I_0 - A_1 e^{-t/\tau_1}$ in the a) and d). Double exponential rise equation $I(t) = I_0 - A_1 e^{-t/\tau_1} - A_2 e^{-t/\tau_2} - A_3 e^{-t/\tau_3}$ in the (b) and (e). And triple exponential rise equation $I(t) = I_0 - A_1 e^{-t/\tau_1} - A_2 e^{-t/\tau_2}$ in the (c) and (f). The fitting parameters are shown in each graph, respectively. All the fittings of the experimental data conducted without imposing any constraints on any value of the fitted parameters.

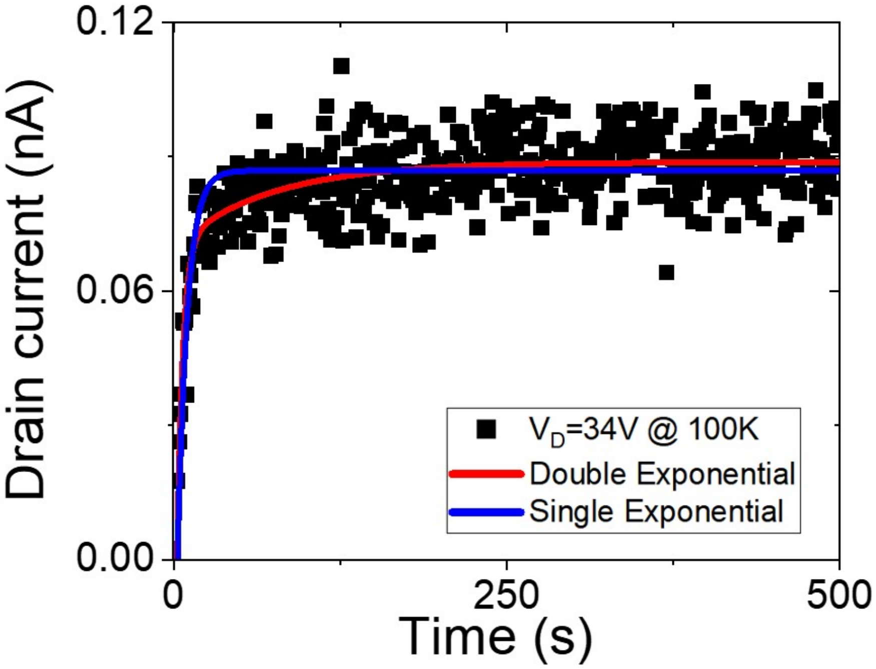

Fig. S14: Plot of the measured (black data points) emission current for $V_D = 34V$ at 100 K and fit using a single (blue) and double (red) rise exponential. The coefficient of determination (R^2) obtained in the fit for the single exponential is 0.6 whereas for the double exponential is of 0.77.

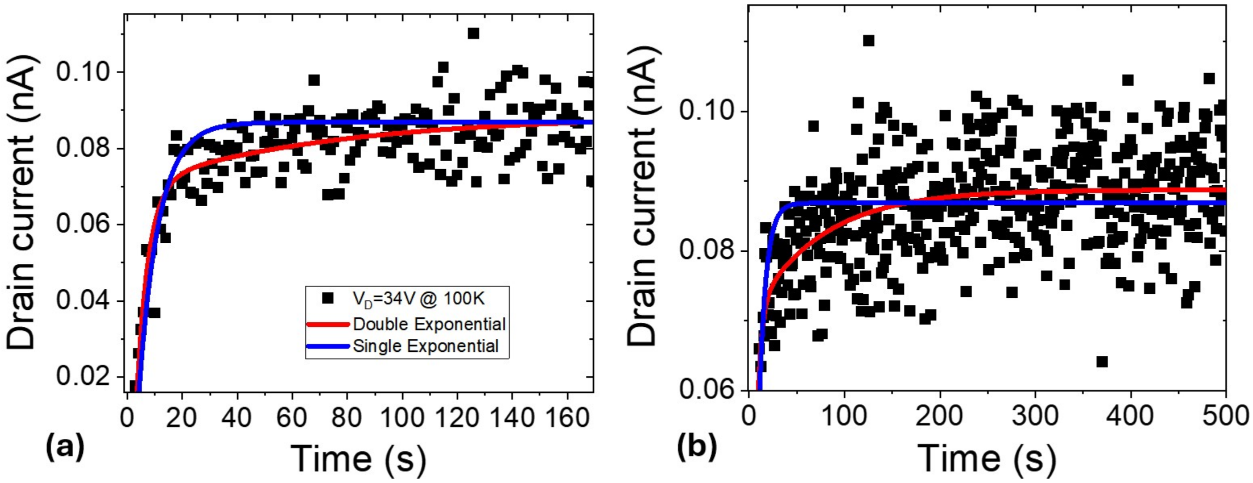

Fig. S15: (a) Zoom of the plot shown in Fig. S10 for a shorter time scale evidencing the better fit by the double exponential in the faster time scale found for the 100 K range. (b Zoom of the plot shown in Fig. S10 for the high range of drain current for a long time scale evidencing that the single exponential fails to describe the overall trend of the data.)

S8. Temperature dependence on the I_{SD} hysteresis under illumination

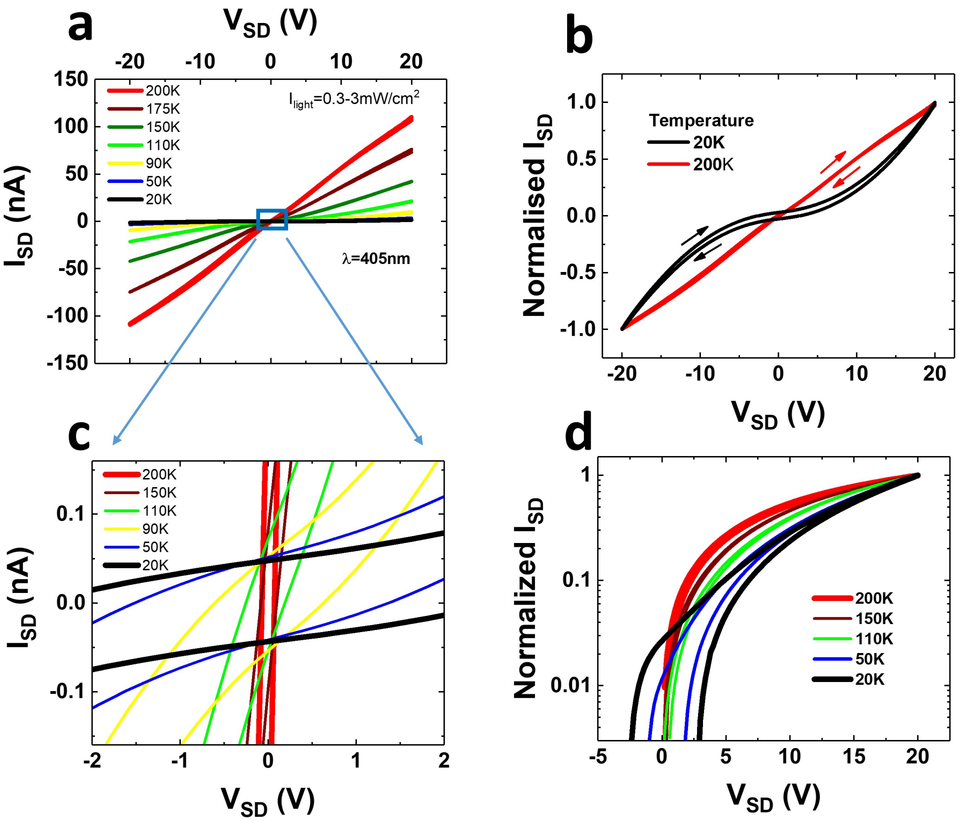

Fig. S16: (a) Plot of the I_{SD} *vs.* V_{SD} measured at various temperatures from 20K to 200K while illuminating with λ = 405nm. (b) Hysteresis of the normalized I_{SD} *vs.* V_{SD} at 20K and 200K. (c) Focused plot of panel (a). I_{SD} *vs.* V_{SD} hysteresis for $V_{SD} = \pm 2V$ measured at various temperatures from 20K to 200K while illuminating with λ = 405nm and is sweeping at the range $V_{SD} = \pm 20V$. (d) Semi-logarithmic scale of the normalized I_{SD} *vs.* V_{SD} hysteresis for 20 K to 200 K and V_{SD} between -5V and 20V while illuminating with λ = 405nm.

Fig. S14 shows up and down sweeps of $I_{SD}(V_{SD})$ at fixed temperatures ranging from 20K up to 200 K upon illumination with the parameters specified in the figure. A small hysteresis is observed at cryogenic temperature, and this is reduced upon increasing the temperature. Prior studies on other pervoskites have reported the role of charges or ion accumulation at the perovskite/metal interface leading to a capacitive hysteresis.[15] In those perovskites, ion

migration increases as the temperature increases leading to an increase in the capacitive hysteresis. This is in stark contrast to our observations, which clearly show a decreasing hysteresis for increasing temperature. Therefore, the observed hysteresis in 2D F-PEAI does not bare an ionic contribution.

S9. Additional IV data under illumination $\lambda = 514nm$, room temperature sublinear functional dependence for $\lambda = 375\,nm$ and emission state.

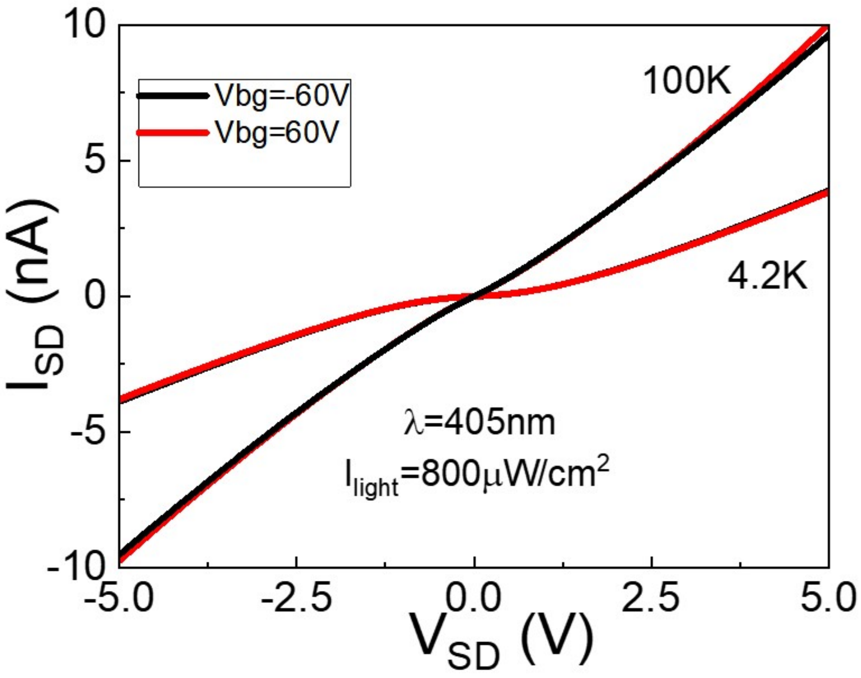

Fig. S17: Plot of the measured I_{SD} *vs.* V_{SD} illuminating the whole photoactive area of the photodetector $A_{act} \approx 19.000\,\mu\text{m}^2$) with light $\lambda = 514\,\text{nm}$ and irradiance $I_{light} = 800\,\mu\text{W}/\text{cm}^2$, and for fixed values of V_{BG} expanding the data set shown in Fig. 4a.

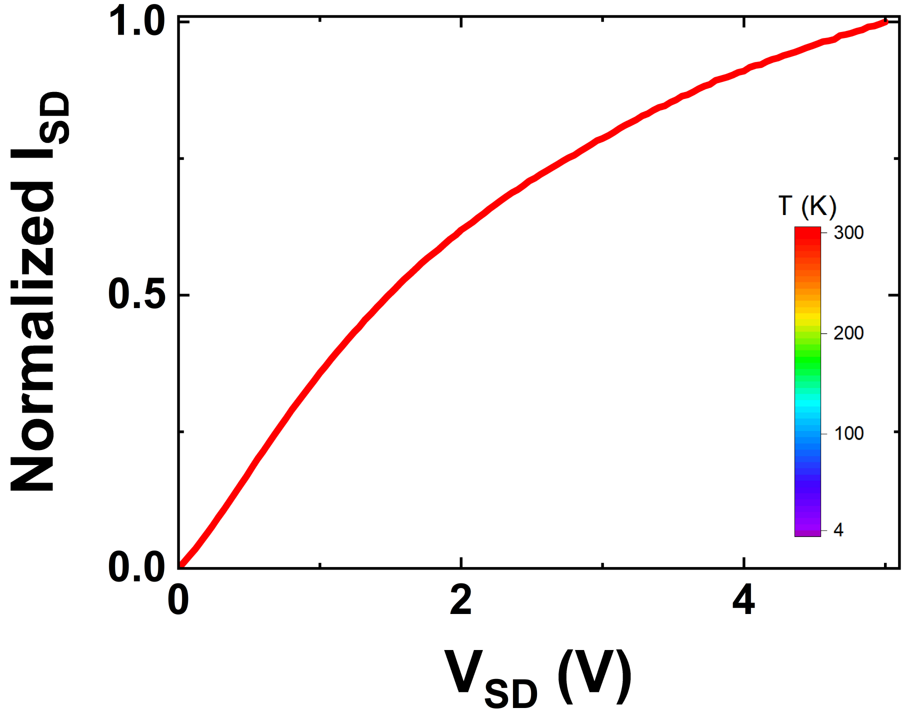

Fig. S18: Plot of the normalized I_{SD} *vs.* V_{SD} measured at room temperature while illuminating with $\lambda = 375\,\text{nm}$ (I_{light}=1 mW/cm^2).

S10. External quantum efficiency

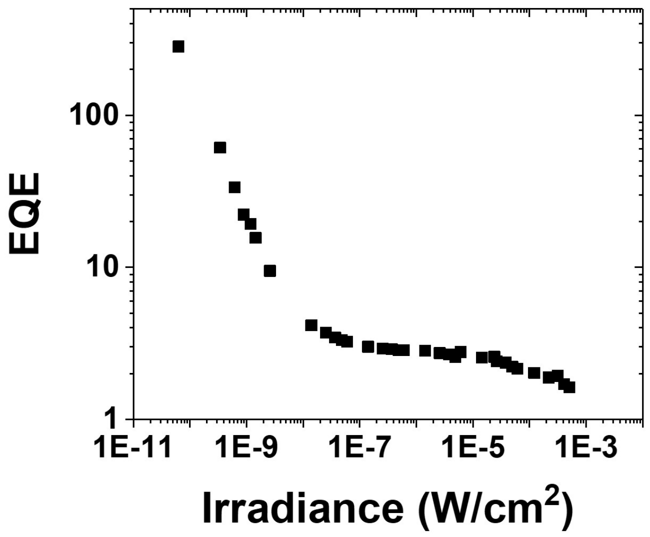

Fig. S19: External quantum efficiency for the 2D F-PEAI photodetector of Fig. 5 in the main manuscript. Similar values of EQE>100% were previously reported in more than 10 2D F-PEAI photodetectors[2]

S11. Noise equivalent power

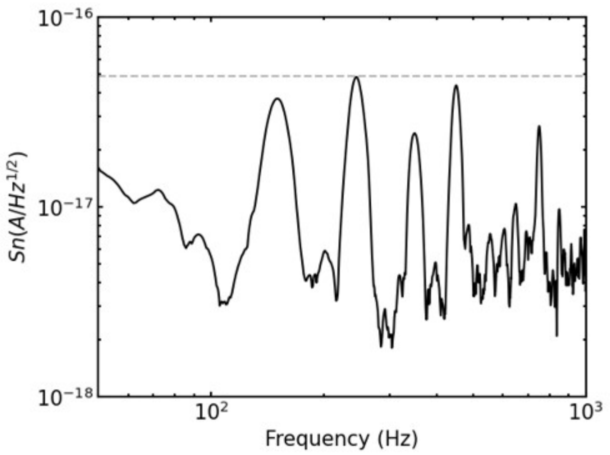

Fig. S20: Noise equivalent power for the 2D F-PEAI photodetector of Fig. 5 in the main manuscript. Similar values of S_n were previously reported in a number of 2D F-PEAI photodetectors[2]

S12. TVTS tolerance to contact resistance

TVTS relies on monitoring the time evolution of the threshold voltage $V_{th}(t)$ in a field-effect transistor following a controlled filling of trap states. The protocol measures the transient shift in threshold voltage rather than the absolute magnitude of the drain current. As shown below, the transient component that carries information about trap emission is unaffected by any constant series resistance, including contact resistance. This is quantitatively demonstrated by modeling the contact resistance (R_C) as a constant resistance in series to the device.[?] In this case, the transient channel current in the linear regime for the transistor shown in Eq.(1) from the main manuscript becomes: $I_d(t) = \frac{\mu \frac{W}{L} C_{ox}[V_g - V_{th}(t)]V_d}{1 + R_C \mu \frac{W}{L} C_{ox}[V_G - V_{th}(t)]}$ where C_{ox} is the capacitance to the back gate. Two limits encompass all realistic devices.

The contact dominated regime where $R_C \gg (\mu \frac{W}{L} C_{ox}(V_g - V_{th}))^{-1}$ resulting in a time independent $I_d \approx \frac{V-d}{R_C}$. In this case, the emission transient disappears from the measured current, because it is entirely masked by the large series resistance.

In the opposite limit, that is $R_C \ll (\mu \frac{W}{L} C_{ox}(V_g - V_{th}))^{-1}$, the expression reduces to the usual FET relation, and the current retains the same exponential form as the ideal case. Since TVTS evaluates the transient part associated with $V_{th}(t)$, the contact resistance only modifies the prefactor in the drain-current expression (or suppresses the current entirely in extreme cases), but it cannot alter the exponential dependence originating from the trap emission in the threshold voltage. Therefore the extracted trap parameters remain independent of the magnitude of R_C. This demonstrates that TVTS provides reliable access to trap dynamics even in devices with significant contact resistance, making this technique suitable to characterise defect states in a wide range of devices without requiring their optimisation.

Trap Characterization Techniques (Table S1)

Table S1 presents a summary of the trap states characterization techniques demonstrated on perovskites to date, starting from TVTS. The compatibility of each method to 2D perovskites and their transistors and devices under operation is also highlighted. The minimal requirements on environmental and operating parameters needed for the characterization are discussed, such as temperature and frequency.

Method	Technique	Measured Parameters	Suitable for (Fabrication stage / Device Geometry)	Contacts / 2D compatibility	Features	Limitations	Ref.
Electrical/DC	Transient threshold voltage spectroscopy (TVTS)	Transient dark current only at one specific temperature (no need for temperature dependent measurements)	Complete device/ Field effect transistor (no need for p-n junction or any diode geometry)	Valid on ohmic and Schottky contacts/ Valid only on 2D channels	-Distinguish deep from shallow traps at a specific temperature (no need for Arrhenius plot)<br>-Can extract the density of the deep traps<br>-Suitable for slow trapped charges (<1mHz)<br>-Can extract the energy of traps with Kelvin probe force microscopy (KPFM)	-Applicable only on 2D channels<br>-No spatial resolution (extract information from the whole area of the channel)	[16,17]

Table S1 – continued from previous page

Method	Technique	Measured Parameters	Suitable for (Fabrication stage / Device Geometry)	Contacts / 2D compatibility	Features	Limitations	Ref.
Electrical/AC	Drive-level capacitance profiling (DLCP)	Temperature dependent steady state capacitance	Complete device / Need of p-n junction or Schottky diode geometry	Only ohmic contacts / No straightforward 2D compatible	- Measures spatial and energetic distribution of trap throughout the perovskite layer<br>- Measures both the shallow and deep defects at the same time<br>- Gives activation energy, and provides higher resolution to defect evaluation than any other techniques	- High low frequency noise (especially when the frequency is lower than 1 kHz)<br>- Complicated setup, difficult to extract data	[18–20]

Table S1 – continued from previous page

Method	Technique	Measured Parameters	Suitable for (Fabrication stage / Device Geometry)	Contacts / 2D compatibility	Features	Limitations	Ref.
Electrical/AC	Deep-level transient spectroscopy (DLTS)	Temperature dependent transient capacitance	Complete device / Need of p-n junction / Schottky diode geometry	Only ohmic contacts / No straightforward 2D compatible	- Highly sensitive for the detection of deep-level defects<br>- Providing information about activation energy and electron/hole capture cross sections	- Cannot observe shallow-level defect traps with high thermal emission rate<br>- Might miss minority carrier traps that cannot be saturated at practical levels of forward current<br>- Suitable only for high conductive materials (no insulators)	[18,21–23]

Table S1 – continued from previous page

**Method**	**Technique**	**Measured Parameters**	**Suitable for (Fabrication stage / Device Geometry)**	**Contacts / 2D compatibility**	**Features**	**Limitations**	**Ref.**
Electrical/DC	Space-charge limited current (SCLC)	Steady state DC current measurements	Complete 2 terminal devices / Only one type of carrier device	Contacts that allow only 1 carrier injection / 2D channel compatible	- Information about two types of defect density, i.e., electron and hole density<br>- Temperature dependent SCLC gives activation energy of defects	- Only one type of defect density at one time can be measured<br>- Deviation in estimated defect density due to difficulty in accurate determination of kink point of VTFL<br>- Need fabrication of two types of an electron as well as hole-only devices	[18,24–26]

Table S1 – continued from previous page

Method	Technique	Measured Parameters	Suitable for (Fabrication stage / Device Geometry)	Contacts / 2D compatibility	Features	Limitations	Ref.
Electrical/AC	Thermal admittance spectroscopy (TAS)	Temperature dependent steady state capacitance (under illumination and no illumination)	Complete device / Need of p-n junctions / Schottky diodes	Ohmic contacts / No straightforward 2D compatible	- Shallow and deep traps can be obtained by tracing the junction capacitance<br>- The activation energy of the defects can be calculated and can be performed on the complete device under dark and illumination	- Unable to distinguish between the valence band and conduction band states<br>- Only defects with the energy below the energy demarcation can contribute to the capacitance signal<br>- Trapped charges with long thermal emission time cannot contribute to the capacitance signal	[18,24,27–29]

Table S1 – continued from previous page

**Method**	**Technique**	**Measured Parameters**	**Suitable for (Fabrication stage / Device Geometry)**	**Contacts / 2D compatibility**	**Features**	**Limitations**	**Ref.**
Opto-Electrical/DC	Thermally stimulated current (TSC)	Temperature dependent steady state current under illumination and no illumination	Complete 2 terminal device / Semi-insulating channel	Ohmic contact / 2D compatible	- A wide range of defect levels can be probed by tracing thermally activated current<br>- Information about the activation energy of defects<br>- Can be performed under illumination on regular working solar cells	- Unable to distinguish between the valence band and conduction band states<br>- Only the lower limit of defect density can be evaluated<br>- Trapped charges having long thermal emission time do not contribute to a thermally activated current signal	[18,30,31]

Table S1 – continued from previous page

**Method**	**Technique**	**Measured Parameters**	**Suitable for (Fabrication stage / Device Geometry)**	**Contacts / 2D compatibility**	**Features**	**Limitations**	**Ref.**
Opto-Electrical/DC	Ultrafast photocurrent spectroscopy (UPS) and photo induced current transient spectroscopy (PICTS)	Temperature dependent transient photocurrent	Complete 2 terminal device / Illumination with LED or laser	Ohmic contacts / 2D compatible	- Eliminates scattering and reflection artifacts giving it an outstanding dynamic range<br>- Sufficient for the identification of very low concentrations of defect states and intermolecular interactions	- Need of temperature dependent measurements for the dynamic characterization of traps<br>- UPS needs fitting with Photon Assisted Tunneling model (PAT) to extract traps concentration, EA, scattering cross section. Many fitting parameters	[18,32,33]

Table S1 – continued from previous page

**Method**	**Technique**	**Measured Parameters**	**Suitable for (Fabrication stage / Device Geometry)**	**Contacts / 2D compatibility**	**Features**	**Limitations**	**Ref.**
Opto-Electrical/DC	Photoinduced charge extraction by linearly increasing the voltage (photo-CELIV) and Time-of-Flight	Temperature dependent transient current under illumination	Complete 2T device / Illumination with LED / laser need of large channel	Ohmic contacts / 2D compatible	- Accurate measurement of charge carrier mobility<br>- Investigation of bulk charge transport in perovskite films	- Cannot distinguish the type of charge carrier<br>- Only qualitative analysis of trap states	[18,34,35]

References

1. Russo, S.; Craciun, M. F.; Yamamoto, M.; Morpurgo, A. F.; Tarucha, S. Contact resistance in graphene-based devices. *Physica E: Low-dimensional Systems and Nanostructures* **2010**, *42*, 677–679.

2. Mastria, R.; Riisnaes, K. J.; Bacon, A.; Leontis, I.; Lam, H. T.; Alshehri, M. A. S.; Colridge, D.; Chan, T. H. E.; De Sanctis, A.; De Marco, L.; Polimeno, L.; Coriolano, A.; Moliterni, A.; Olieric, V.; Giannini, C.; Hepplestone, S.; Craciun, M. F.; Russo, S. Real Time and Highly Sensitive Sub-Wavelength 2D Hybrid Perovskite Photodetectors. *Advanced Functional Materials* **2024**, *2401903*, 1–9.

3. Intonti, K.; Faella, E.; Viscardi, L.; Kumar, A.; Durante, O.; Giubileo, F.; Passacantando, M.; Lam, H. T.; Anastasiou, K.; Craciun, M.; Russo, S.; Di Bartolomeo, A. Hysteresis and Photoconductivity of Few-Layer ReSe2 Field Effect Transistors Enhanced by Air Pressure. *Advanced Electronic Materials* **2023**, *9*, 2300066.

4. Durante, O.; Intonti, K.; Viscardi, L.; De Stefano, S.; Faella, E.; Kumar, A.; Pelella, A.; Romeo, F.; Giubileo, F.; Alghamdi, M. S. G.; Alshehri, M. A. S.; Craciun, M. F.; Russo, S.; Antonio, D. B. Subthreshold Current Suppression in ReS2 Nanosheet-Based Field-Effect Transistors at High Temperatures. *ACS Applied Nano Materials* **2023**, *6*, 21663–21670.

5. Mastria, R.; Riisnaes, K. J.; Bacon, A.; Leontis, I.; Lam, H. T.; Alshehri, M. A. S.; Colridge, D.; Chan, T. H. E.; De Sanctis, A.; De Marco, L.; Polimeno, L.; Coriolano, A.; Moliterni, A.; Olieric, V.; Giannini, C.; Hepplestone, S.; Craciun, M. F.; Russo, S. Real Time and Highly Sensitive Sub-Wavelength 2D Hybrid Perovskite Photodetectors. *Advanced Functional Materials* *n/a*, 2401903.

6. Menahem, M.; Dai, Z.; Aharon, S.; Sharma, R.; Asher, M.; Diskin-Posner, Y.; Ko-

robko, R.; Rappe, A. M.; Yaffe, O. Strongly Anharmonic Octahedral Tilting in Two-Dimensional Hybrid HalidePerovskites. *ACS Nano* **2021**, *15*, 1015310162.

7. Shihara, T.; Takahashi, J.; Goto, T. Optical properties due toelectronic transitions in two-dimensional semiconductors (CnH2n+1NH3)2PbI4. *Phys. Rev. B* **1990**, *42*, 1109911107.

8. Martín-García, B.; Spirito, D.; Biffi, G.; Artyukhin, S.; Bonaccorso, F.; Krahne, R. Phase Transitions in Low-DimensionalLayered Double Perovskites: The Role of the Organic Moieties. *J.Phys. Chem. Lett.* **2021**, *12*, 280286.

9. Li, M.-K.; Chen, T.-P.; Lin, Y.-F.; Raghavan, C. M.; Chen, W.-L.; Yang, S.-H.; Sankar, R.; Luo, C.-W.; Chang, Y.-M.; Chen, C.-W. Intrinsic Carrier Transport of Phase-Pure Homologous 2D Organolead Halide Hybrid Perovskite Single Crystals. *Small* **2018**, *14*, 1803763.

10. Ziegler, J. D.; Lin, K.-Q.; Meisinger, B.; Zhu, X.; Kober-Czerny, M.; Nayak, P. K.; Vona, C.; Taniguchi, T.; Watanabe, K.; Draxl, C.; Snaith, H. J.; Lupton, J. M.; Egger, D. A.; Chernikov, A. Excitons at the Phase Transition of 2D Hybrid Perovskites. *ACS Photonics* **2022**, *9*, 36093616.

11. Dey, B.; Islam, M. S.; Pervin, U.; Al Mamun Mazumder, A.; Makino, T.; Park, J. Temperature-induced localized exciton dynamics in inorganic CsPbX3 (X=I, Br, Cl) perovskite nanocrystals. *Journal of Luminescence* **2025**, *281*, 121199.

12. Sarkar, S.; Kamath, N. S.; Gayen, K.; Pal, S. K. Exciton-phonon coupling in quasi-two-dimensional Ruddlesden-Popper perovskites: impact of a mixed-phase structure. *Nanoscale* **2025**, *17*, 10771–10783.

13. Perdew, J. P.; Burke, K.; Ernzerhof, M. Generalized gradient approximation made simple. *Physical Review Letters* **1996**, *77*, 3865–3868.

14. Bube, R. H. *Cambridge University Press*; 1992.

15. Almora, O.; Aranda, C.; Zarazua, I.; Guerrero, A.; Garcia-Belmonte, G. Noncapacitive Hysteresis in Perovskite Solar Cells at Room Temperature. *ACS Energy Letters* **2016**, *1*, 209–215.

16. Amit, I.; Octon, T. J.; Townsend, N. J.; Reale, F.; Wright, C. D.; Mattevi, C.; Craciun, M. F.; Russo, S. Role of Charge Traps in the Performance of Atomically Thin Transistors. *Advanced Materials* **2017**, *29*, 1605598.

17. Townsend, N. J.; Amit, I.; Panchal, V.; Kazakova, O.; Craciun, M. F.; Russo, S. Energy dispersive spectroscopic measurement of charge traps in MoTe2. *Physical Review B* **2019**, *100*, 165310.

18. Bao, C.; Gao, F. Physics of defects in metal halide perovskites. *Reports on Progress in Physics* **2022**, *85*, 096501.

19. Ni, Z.; Bao, C.; Liu, Y.; Jiang, Q.; Wu, W.-Q.; Chen, S.; Dai, X.; Chen, B.; Hartweg, B.; Yu, Z.; Holman, Z.; Huang, J. Resolving spatial and energetic distributions of trap states in metal halide perovskite solar cells. *Science* **2020**, *367*, 1352–1358.

20. Zhang, X.; Ma, Y.; Zhang, Z.; Zhang, X.; Yan, H.; Chen, X.; Zheng, Z.; Zhang, Y. The resolutions of drive-level capacitance profiling technique. *Review of Scientific Instruments* **2023**, *94*, 065109.

21. Yang, W. S.; Park, B.-W.; Jung, E. H.; Jeon, N. J.; Kim, Y. C.; Lee, D. U.; Shin, S. S.; Seo, J.; Kim, E. K.; Noh, J. H.; Seok, S. I. Iodide management in formamidinium-lead-halide–based perovskite layers for efficient solar cells. *Science* **2017**, *356*, 1376–1379.

22. Zhao, Y.; Tripathi, M.; Čerņevičs, K.; Avsar, A.; Ji, H. G.; Marin, J. F. G.; Cheon, C.-Y.; Wang, Z.; Yazyev, O. V.; Kis, A. Electrical spectroscopy of defect states and their hybridization in monolayer MoS2. *Nature Communications* **2023**, *14*, 44.

23. Ciavatti, A.; Foderà, V.; Armaroli, G.; Maserati, L.; Colantoni, E.; Fraboni, B.; Cavalcoli, D. Adv. Funct. Mater. *Advanced Functional Materials* **2024**, *34*, 2405291.

24. Shi, D.; Adinolfi, V.; Comin, R.; Yuan, M.; Alarousu, E.; Buin, A.; Chen, Y.; Hoogland, S.; Rothenberger, A.; Katsiev, K.; Losovyj, Y.; Zhang, X.; Dowben, P. A.; Mohammed, O. F.; Sargent, E. H.; Bakr, O. M. Low trap-state density and long carrier diffusion in organolead trihalide perovskite single crystals. *Science* **2015**, *347*, 519–522.

25. Dong, Q.; Fang, Y.; Shao, Y.; Mulligan, P.; Qiu, J.; Cao, L.; Huang, J. Electron-hole diffusion lengths > 175 μm in solution-grown CH₃NH₃PbI₃ single crystals. *Science* **2015**, *347*, 967–970.

26. Adinolfi, V.; Yuan, M.; Comin, R.; Thibau, E. S.; Shi, D.; Saidaminov, M. I.; Kanjanaboos, P.; Kopilovic, D.; Hoogland, S.; Lu, Z.-H.; Bakr, O. M.; Sargent, E. H. The In-Gap Electronic State Spectrum of Methylammonium Lead Iodide Single-Crystal Perovskites. *Advanced Materials* **2016**, *28*, 3406–3410.

27. Shao, Y.; Xiao, Z.; Bi, C.; Yuan, Y.; Huang, J. Origin and elimination of photocurrent hysteresis by fullerene passivation in $CH_3NH_3PbI_3$ planar heterojunction solar cells. *Nature Communications* **2014**, *5*, 5784.

28. Lin, Y.; Chen, B.; Zhao, F.; Zheng, X.; Deng, Y.; Shao, Y.; Fang, Y.; Bai, Y.; Wang, C.; Huang, J. Matching Charge Extraction Contact for Wide-Bandgap Perovskite Solar Cells. *Advanced Materials* **2017**, *29*, 1700607.

29. Lin, Y.; Shen, L.; Dai, J.; Deng, Y.; Wu, Y.; Bai, Y.; Zheng, X.; Wang, J.; Fang, Y.; Wei, H.; Ma, W.; Zeng, X. C.; Zhan, X.; Huang, J. -Conjugated Lewis Base: Efficient Trap-Passivation and Charge-Extraction for Hybrid Perovskite Solar Cells. *Advanced Materials* **2017**, *29*, 1604545.

30. Baumann, A.; Väth, S.; Rieder, P.; Heiber, M. C.; Tvingstedt, K.; Dyakonov, V. Identification of Trap States in Perovskite Solar Cells. *The Journal of Physical Chemistry Letters* **2015**, *6*, 2350–2354.

31. Hu, Y.; Hutter, E. M.; Rieder, P.; Grill, I.; Hanisch, J.; Aygüler, M. F.; Hufnagel, A. G.; Handloser, M.; Bein, T.; Hartschuh, A.; Tvingstedt, K.; Dyakonov, V.; Baumann, A.; Savenije, T. J.; Petrus, M. L.; Docampo, P. Understanding the Role of Cesium and Rubidium Additives in Perovskite Solar Cells: Trap States, Charge Transport, and Recombination. *Advanced Energy Materials* **2018**, *8*, 1703057.

32. Kobbekaduwa, K.; Shrestha, S.; Adhikari, P.; Liu, E.; Coleman, L.; Zhang, J.; Shi, Y.; Zhou, Y.; Bekenstein, Y.; Yan, F.; Rao, A. M.; Tsai, H.; Beard, M. C.; Nie, W.; Gao, J. In-situ observation of trapped carriers in organic metal halide perovskite films with ultra-fast temporal and ultra-high energetic resolutions. *Nature Communications* **2021**, *12*, 1636.

33. Thiesbrummel, J.; Le Corre, V. M.; Peña-Camargo, F.; Perdigón-Toro, L.; Lang, F.; Yang, F.; Grischek, M.; Gutierrez-Partida, E.; Warby, J.; Farrar, M. D.; Mahesh, S.; Caprioglio, P.; Albrecht, S.; Neher, D.; Snaith, H. J.; Stolterfoht, M. Universal Current Losses in Perovskite Solar Cells Due to Mobile Ions. *Advanced Energy Materials* **2021**, *11*, 2101447.

34. Musiienko, A.; Pipek, J.; Praus, P.; Brynza, M.; Belas, E.; Dryzhakov, B.; Du, M. H.; Ahmadi, M.; Grill, R. Deciphering the effect of traps on electronic charge transport properties of methylammonium lead tribromide perovskite. *Science Advances* **2020**, *6*.

35. Stephen, M.; Genevičius, K.; Juška, G.; Arlauskas, K.; Hiorns, R. C. Charge transport and its characterization using photo-CELIV in bulk heterojunction solar cells. *Polymer International* **2017**, *66*, 13–25.
